# Transcriptomic and Functional Analysis of NaCl-Induced Stress in *Enterococcus faecalis*

**DOI:** 10.1371/journal.pone.0094571

**Published:** 2014-04-22

**Authors:** Margrete Solheim, Sabina Leanti La Rosa, Thomas Mathisen, Lars G. Snipen, Ingolf F. Nes, Dag Anders Brede

**Affiliations:** 1 Laboratory of Microbial Gene Technology and Food Microbiology, The Norwegian University of Life Sciences, Ås, Norway; 2 Section for Biostatistics, Department of Chemistry, Biotechnology and Food Science, The Norwegian University of Life Sciences, Ås, Norway; University of Liverpool, United Kingdom

## Abstract

The robust physiology of *Enterococcus faecalis* facilitates tolerance to various stresses. We here report the transcriptional response of *E. faecalis* V583 to growth in the presence of 6.5% NaCl. Among the early responses observed was an immediate down-regulation of *mscL*, accompanied by an up-regulation of genes predicted to be involved in uptake of extracellular potassium and glycine betaine. The high NaCl concentration also induced expression of chaperons and cell envelope related traits, such as the enterococcal polysaccharide antigen (*epa*) locus. Functional genetic analysis revealed reduced salt stress resistance in both *epaB* and *epaE* mutants. The reduced salt resistance phenotype associated with the *epaB* mutant was restored by complementation, hence demonstrating a role of Epa in the physiological robustness of *E. faecalis*. Furthermore, we demonstrate that Epa confers increased resistance towards multiple cell envelope stress-inducing factors. Accordingly, these findings delineate a potential link between the robust nature of *E. faecalis* and its ability to perform as a human pathogen, and provide a new perspective on the mechanisms by which Epa contributes to virulence. Notably, the high NaCl concentration also resulted in strict repression of the *gelE-sprE* operon and impaired gelatinase activity. We demonstrate that NaCl antagonize the GBAP-pheromone dependent induction in a concentration dependent manner.

## Introduction

*Enterococcus faecalis* constitutes part of the normal intestinal flora of humans, and only sporadic reports of enterococcal infections in immunocompromised patients existed until the 1980s [1]. In recent years however, *E. faecalis* has emerged as a clinical important opportunistic pathogen. Enterococci now rank among the leading causes of nosocomial infections worldwide [2], [3]. Medical treatment is difficult, as enterococci, favored by a high conjugation rate, have acquired resistance mechanisms against the most commonly used antibiotics [4].

Generally, the *E. faecalis* species challenges the boundary between commensal and pathogen: while several genetic traits that contribute to the virulence of *E. faecalis* have been characterized (reviewed in [5]), none has appeared to be indispensable for its pathogenicity. A distinct trait in *E. faecalis* physiology, compared to other intestinal lactic acid bacteria, is its ability to persist and thrive in harsh environments, that include heat, acid, oxidative and hyperosmotic stress [6]. It is thus conceivable that the intrinsic robustness of *E. faecalis* is significant to the pathogenic potential of this bacterium. In this context, acquiring in-depth knowledge of the basic physiology of *E. faecalis* as well as exploring the specific traits that enable this bacterium to persist is imperative in the quest to understand *E. faecalis* pathogenicity.

Elevated osmolarity is among the many stressful conditions encountered by this bacterium in its natural habitat, *e.g.* the salinity of the small intestines is equivalent to 0.3 M NaCl. Interestingly, it was recently demonstrated that mechanisms involved in intrinsic resistance to osmotic stress were major constituents to multidrug resistance in *Acinetobacter baumannii*, and may thus contribute to the persistence of this emerging nosocomial pathogen in clinical settings [7]. Previous reports suggest that enterococci control turgor by actively modulating the pool of osmotically active solutes in their cytoplasm, thereby allowing water content to be adjusted by osmosis [8], [9]. As part of a continued effort to decipher the various physiological aspects contributing to the success of this versatile pathogen, we here describe the global transcriptional profile of *E. faecalis* V583 upon the encounter with high concentrations of NaCl.

## Materials and Methods

### Bacterial Strain and Growth Conditions

Bacterial strains and plasmids used in this study are listed in Table 1. *E. faecalis* strains were grown as previously described [10]. NaCl were solubilized in water to obtain 5 M solution. Autoclaved stock solution was added to autoclaved medium. Antibiotic concentrations (per ml) were: 10 µg erythromycin, 12.5 µg chloramphenicol, 12.5 µg tetracycline and 150 µg spectinomycin for *Escherichia coli* and 15 µg erythromycin, 25 µg tetracycline and 500 µg spectinomycin for *E. faecalis*.

**Table 1:** Bacterial strains and plasmids used in this study.

Strain or plasmid	Characteristic(s)	Reference
Strains		
* E. coli*		
GeneHogs		Invitrogen
EPI300		Epicentre
* E. faecalis*		
V583		[58]
OG1RF		[59]
TX5179	OG1RF Δ*epaB*	[41]
TX5180	OG1RF Δ*epaE*	[41]
OU510	Contains an amber mutation in *fsrD*, resulting in a lack of GBAP biosynthesis	[20]
LMGT3690	V583Δ*gelE*	Diep, Hernandez and Nes, unpublished
MS232	V583 pAT28p11*fsrD*	This study
MS234	V583Δ*gelE* pAT28p11*fsrD*	This study
MS253	OU510 empty pAT28	This study
MS269	OU510 pREG696-P*_fsrB_*-*luxABCDE*	This study
MS272	OU510 pREG696-P*_gelE_*-*luxABCDE*	This study
MS377	TX5179 pAT28*epaBCD* with native promoter	This study
MS381	TX5179 empty pAT28	This study
MS383	OG1RF empty pAT28	This study
Plasmids		
pCC1	Single-copy cloning vector, cam^R^	Epicentre
pAT28	Shuttle vector, spec^R^	[13]
pAT28pCC1 *efaBCD*	*efaBCD* and their native promoter inserted in pAT28	This study
pAT28 P_11_ *fsrD*	*fsrD* and the P_11_ promoter inserted in pAT28	This study
pLei1		Leanti La Rosa, unpublished
pREG696	Low-copy-number vector, stable due to *axe-txe* toxin-antitoxin locus, spec^R^	[18]
pREG696-P*_fsrB_*-*luxABCDE*		This study
pREG696-P*_gelE_*-*luxABCDE*		This study
pSL101P*_16S_*	pPL2*lux* derivative containing a synthetic P16S promoter and the *axe-txe*cassette, spec^R^	[19]

Amp = ampicillin, cam = cloramphenicol, ery = erythromycin, r = resistance and spec = spectinomycin.

### NaCl Treatment

For broth assays, overnight (ON) cultures were inoculated (50× dilution) into BHI, containing various concentrations of NaCl. Cell growth was measured spectrophotometrically and by viable cell counts as previously described [11]. The added NaCl concentrations ranged between 1–8% (inherent amounts of NaCl in BHI have not been counted in). All experiments were performed independently in triplicates.

### Sample Collection

ON cultures were diluted 50× and grown in BHI to an OD_600_ of ∼ 0.2 and split into two. 5 M NaCl was added to one of the cultures, to a final concentration of 6.5% NaCl. An equal volume of sterile H_2_O was added to the second culture (control culture), to neutralize the dilution factor. The two cultures were then further incubated, and 10 mL samples were collected immediately after addition of NaCl (*t_5_*), and then after 30 (*t_30_*) and 60 min (*t_60_*). Samples were centrifuged at 5000 rpm for 5 min in an Eppendorf 5804R tabletop centrifuge at 4°C, and pellets were flash frozen in N_2_ (*l*) prior to RNA extraction.

### RNA Isolation, cDNA Synthesis, Fluorescent Labeling, Hybridization and Data Analysis

Total RNA was isolated by FastPrep (Bio 101/Savant) and RNeasy Mini kit (QIAGEN) as previously described [11]. The concentrations of the RNA samples were measured by using the NanoDrop (NanoDrop Technologies), and the quality was assessed by using the RNA 600 Nano LabChip kit and the Bioanalyzer 2100 (Agilent Technologies). cDNA was synthesized and labeled with the Fairplay II Microarray labeling kit (Stratagene), with modifications as previously described [11]. Labeled samples were then dried, prior to resuspension in 140 µl hybridization solution and hybridized as described by Vebø et al. [10]. The microarray used in this work has also been described previously [12]. Three replicate hybridizations were performed with three separate batches of RNA. The three batches of RNA were obtained in three separate growth experiments. The Cy3 and Cy5 dyes (Amersham) used during cDNA synthesis were swapped in one of the three replicate hybridizations. All samples were co-hybridized with control samples collected at equal time points (*e.g.* t_30_ was hybridized along with t_30_). Hybridized arrays were scanned at wavelengths of 532 nm (Cy3) and 635 nm (Cy5) with a Tecan scanner LS (Tecan). Fluorescent intensities and spot morphologies were analyzed using GenePix Pro 6.0 (Molecular Devices), and spots were excluded based on slide or morphology abnormalities. Downstream analysis was carried out using the LIMMA package (www.bioconductor.org) in the R computing environment (www.r-project.org) as previously described [10]. Log_2_-ratios are presented as log_2_ (treated/untreated).

### Microarray Data Accession Number

The microarray data have been deposited in the ArrayExpress database with the series accession number E-TABM-904.

### Validation of Microarray Data by Real Time qRT-PCR

Real time quantitative PCR (QPCR) was used to validate the expression levels for the following genes as previously described [10]: EF0282, EF1211 and EF2642 at *t_60_*. *dnaB* was used as a reference. All genes were quantified in triplicate. The analysis was performed on the same batches of RNA as used for the microarray experiments. The primers used are shown in Table S1.

### Complementation of an *E. faecalis* TX5179 Insertion Mutant

Plasmid DNA was extracted with the Qiaprep Spin Miniprep kit and the Qiagen Plasmid Midi kit (QIAGEN) according to the manufacture’s protocol. A complementation construct of TX5179 was made in pAT28 [13]. The *efaBCD* genes and their native promoter were first amplified from OG1RF with epaBpro-F/epaD-R and ligated blunt into the pCC1™ vector (Epicentre). pCC1epaBCD was then digested with EcoRI and subcloned into pAT28, using the EcoRI restriction site. The construct was propagated in *E. coli* EPI300 (Epicentre) and integrity confirmed by DNA sequencing prior to transfer into *E. faecalis*. *E. faecalis* electrocompetent cells were prepared as described by Holo and Nes [14], with 3.5 to 6% glycine in the growth medium. Primers used are listed in Table S1.

### Determination of Minimal Inhibitory Concentration

In order to identify phenotypes in which the enterococcal polysaccharide antigen (Epa) is involved, minimal inhibitory concentration (MIC) of various biologically relevant stressors were determined for wild type OG1RF and two different *epa* mutants (TX5179 and TX5180) by plate assays as previously described [15]. ON cultures (∼10^9^ cells/mL) were diluted 1000× and incubated for 24 h at 37°C. MIC_50_ was defined as the lowest concentration of the stressor that reduced bacterial growth by >50% in mid exponential growth compared to control conditions (untreated cells). The growth of wild type cells were here compared to the growth of untreated wild type, while mutant growth was compared with untreated cells of the relevant mutant. The following compounds were tested: the antibiotics ampicillin and vancomycin, the bacteriocins leucocin A, leucocin C and pediocin PA-1, ethanol, hydrogen peroxide (H_2_O_2_), sodium dodecyl sulphate (SDS) and sodium taurodeoxycholate.

### Transmission Electron Microscopy

To assess phenotypic variations associated with high NaCl concentrations, *E. faecalis* cells growth in BHI with or without the addition of 6.5% NaCl were examined by transmission electron microscopy. Cells for microscopy were collected in mid exponential phase of growth (OD_600_ 0.3–0.4) and washed with PBS buffer. Samples were then prepared by mounting the cells onto a copper grid, followed by immersion in filtered 2% phosphotungstic acid (PTA; pH 7.0) and drying at room temperature.

### Construction of Pheromone Overproducing *E. faecalis* Clones

*fsrD* was amplified from V583 using primers fsrD-F/fsrD-R, digested with XhoI and FatI subcloned into pAT28 containing the synthetic promoter p11 [16] using the XhoI and NcoI cloning sites (see below for details on construction of pAT28P_11_ *sgfp* which was used as a basis for this cloning). The construct was propagated in *E. coli* GeneHogs and integrity confirmed by DNA sequencing prior to transfer into *E. faecalis* V583 and LMGT3960. Primer sequences are listed in Table S1.

### Gelatinase Assays

Gelatinase assays were performed on Todd Hewitt (TH) agar plates supplemented with 3% gelatin, as previously described [12]. 6.5% NaCl was added to the plates when appropriate. Soft agar plug assays were also performed as follows: two cylindrical agar plugs (diameter 8 mm) were removed from a TH agar plate supplemented with 3% gelatin, 500 µg spectinomycin and varying concentrations of NaCl using the large end of a sterile tip from an Eppendorf pipette. The distance between the resulting pockets were ∼5 mm. One of the pockets was subsequently filled in with overnight culture of MS253, diluted 100× in TH soft agar. To the second pocket, 1∶1 TH soft agar and sterile filtrated supernatant from overnight culture of MS234, a V583 Δ*gelE* mutant carrying a constitutively expressed version of *fsrD* for overproduction of pheromone, or sterile TH soft agar (control) was added. Induction of gelatinase activity in MS253 was then monitored as described above. A gelatinase negative pheromone producer was used to exclude an effect of carryover of active proteinase.

To assess the effect of osmotic stress on the gelatinase promoter activity, the *fsrB* and *gelE* promoters were fused to the *lux* cassette from pPL2*lux* [17] via the luciferase reporter system pLei1 (Leanti La Rosa, unpublished). The *fsrB* and *gelE* promoters were cloned into pLei1, as follows: Chromosomal DNA from *E. faecalis* V583 was used as the template in PCRs using primers fsrBpro-F/fsrBpro-R and gelEpro-F/gelEpro-R. The resulting ∼0.5-kb PCR products harbored the regions immediately upstream of *fsrB* and *gelE*, respectively. Notably, the start codons of both genes were included at the ultimate 3′ end of the reverse primers. The P*_gelE_* and P*_fsrB_* amplicons were then digested with XhoI and cloned into SwaI-SalI-digested pLei1, yielding pLei1-P*_gelE_* and pLei1-P*_fsrB_*, respectively. As chromosomal integration of the pLei1 derivatives were not successful, P*_gelE_*-*luxABCDE* and P*_fsrB_*-*luxABCDE* were subsequently excised and ligated into pREG696 [18] as described by Leanti La Rosa et al. [19]. The pREG696-P*_gelE_*-*luxABCDE* and pREG696-P*_fsrB_*-*luxABCDE* constructs were propagated in *E. coli* GeneHogs and integrity confirmed by DNA-sequencing prior to transfer into *E. faecalis* OU510, a clinical isolate having an amber point mutation at the chromosomal *fsrB* codon corresponding to Leu-65 which causes the loss of GBAP production and leads to the gelatinase-negative phenotype [20]. Quantitative measurements of *fsrB* and *gelE* expression during growth in the presence of sterile filtrated supernatant from ON culture of MS232 and various concentration of NaCl were then obtained by bioluminescence analysis using a Xenogen IVIS Lumina II system. Pheromone and NaCl was added to actively growing cells (OD_600_ ∼ 0.2), and bioluminescence monitored in microtiter plates for 10 h. NaCl concentrations 0–4% were used.

## Results and Discussion

### The Growth of *E. faecalis* V583 at High Osmolarity

**Figure 1:**
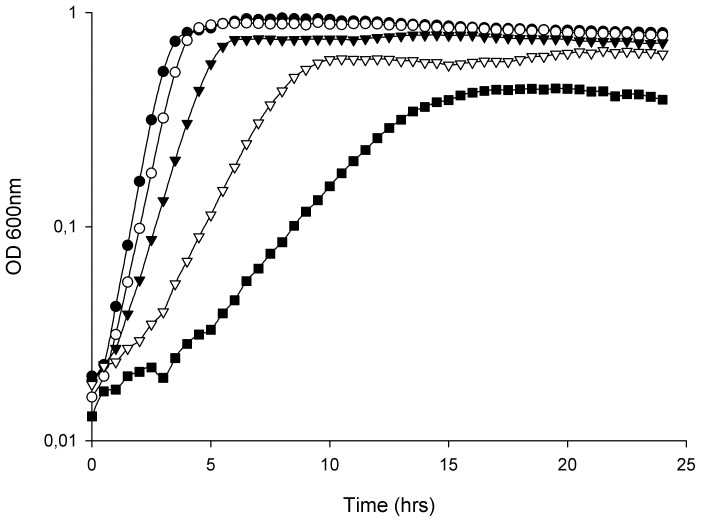
Growth of *E. faecalis* V583 treated with different concentrations of NaCl. Growth of *E. faecalis* V583 treated with different concentrations of NaCl; untreated (•), 2 (○), 4 (▴), 6.5 (Δ) and 8% (▪). All the values are the means from three independent experiments.

The growth of *E. faecalis* V583 at high osmolarity was investigated in BHI broth containing up to 8% NaCl (Figure 1). Increasing concentrations of NaCl led to an extended lag phase, in addition to reduced growth rates and lower cell densities in the stationary phase of growth. The final OD_600_ of V583 challenged with 8% NaCl was approximately half of the final cell density of untreated cultures (Figure 1). Viable cell counts of V583 treated with selected concentrations of NaCl at different time points during growth confirmed the results obtained by OD measurements (results not shown). In general, the NaCl-induced effects on growth reported here, were consistent with the effects of osmotic stress reported in other Gram-positive bacteria [21], [22].

### Transcriptional Response to the Presence of 6.5% NaCl

A genome-scale time course microarray experiment was carried out to characterize the acute transcriptional response of *E. faecalis* V583 to elevated osmolarity (6.5% NaCl). By using a log_2_-ratio >1 and FDR <0.01 cut off criteria, 515 genes were identified as differentially transcribed at one or more time points during the time course of the experiment (Table S2), of which seven genes showed differential expression at all time points (Figure S1 and Table 2). The alkyl hydroperoxide reductase gene *ahpC* (EF2739) was the only genes which was consistently up-regulated throughout the time course. *ahpC* homologs have also previously been linked to osmotic stress responses in other bacteria [23], [24]. Reduced transcription was observed for the EF0633 to -36 operon involved in tyramine production in *E. faecalis* [25]. The production of various amines by decarboxylation of amino acids has also previously been reported to be inhibited by NaCl [26]. In addition, three genes with unknown functions (EF2214, EF2547 and EF3287) and a formate/nitrite transporter family protein (EF0094) were consistently regulated at all three time points.

**Table 2:** Log_2_ ratios for a selection of differentially expressed genes.

Gene product	Cellular role	ORF	Log_2_-ratio at:		
			T(5)	T(30)	T(60)
dTDP-glucose 4,6-dehydratase	Cell envelope	EF2192	0,42	**1,11**	0,37
Glucose-1-phosphate thymidylyltransferase	Cell envelope	EF2194	0,30	**0,98**	0,03
Glycosyl transferase, group 2 family protein	Cell envelope	EF2195	0,69	**1,60**	0,44
Glycosyl transferase, group 2 family protein	Cell envelope	EF2196	0,71	**1,72**	0,25
Glycosyl transferase, group 2 family protein	Cell envelope	EF2197	0,59	**1,90**	0,79
Alkyl hydroperoxide reductase, C subunit	Cellular processes	EF2739	**1,08**	**1,29**	**1,03**
Large conductance mechanosensitive channel protein	Cellular processes	EF3152	**−1,03**	**−**0,63	0,96
NADH peroxidase	Energy metabolism	EF1211	**−**0,55	**2,39**	**4,42**
V-type ATPase, subunit F	Energy metabolism	EF1492	**−**0,41	**2,83**	**1,33**
V-type ATPase, subunit I	Energy metabolism	EF1493	0,03	**2,85**	**1,64**
V-type ATPase, subunit K	Energy metabolism	EF1494	0,32	**3,14**	**1,74**
V-type ATPase, subunit C	Energy metabolism	EF1496	0,21	**3,21**	**2,05**
V-type ATPase, subunit G	Energy metabolism	EF1497	0,38	**2,83**	**1,79**
V-type ATPase, subunit A	Energy metabolism	EF1498	0,33	**3,10**	**1,76**
V-type ATPase, subunit B	Energy metabolism	EF1499	0,46	**3,35**	**2,52**
V-type ATPase, subunit D	Energy metabolism	EF1500	0,24	1,98	**1,53**
Enoyl -(acyl-carrier-protein) reductase	Fatty acid and phospholipid metabolism	EF0282	**−**0,56	**−2,95**	**−1,67**
3-oxoacyl -(acyl-carrier-protein) reductase	Fatty acid and phospholipid metabolism	EF0283	**−**0,17	**−2,11**	**−2,44**
(3R)- -(acyl-carrier-protein) reductase	Fatty acid and phospholipid metabolism	EF0284	**−**0,23	**−1,99**	**−**1,93
Acetyl-CoA carboxylase, carboxyl transferase alpha subunit	Fatty acid and phospholipid metabolism	EF2875	0,04	**−2,24**	**−1,49**
Acetyl-CoA carboxylase, carboxyl transferase beta subunit	Fatty acid and phospholipid metabolism	EF2876	**−**0,03	**−2,58**	**−2,72**
Acetyl-CoA carboxylase, biotin carboxylase	Fatty acid and phospholipid metabolism	EF2877	**−**0,10	**−2,51**	**−2,34**
(3R)-hydroxymyristoyl-(acyl-carrier-protein) dehydratase	Fatty acid and phospholipid metabolism	EF2878	**−**0,18	**−2,31**	**−**1,97
Acetyl-CoA carboxylase, biotin carboxyl carrier protein	Fatty acid and phospholipid metabolism	EF2879	**−**0,28	**−**2,24	**−1,80**
3-oxoacyl-(acyl-carrier-protein) synthase II	Fatty acid and phospholipid metabolism	EF2880	0,31	**−1,94**	**−2,03**
Malonyl CoA-acyl carrier protein transacylase	Fatty acid and phospholipid metabolism	EF2882	0,21	**−2,47**	**−1,71**
Enoyl-(acyl-carrier-protein) reductase II	Fatty acid and phospholipid metabolism	EF2883	**−**0,08	**−3,35**	**−2,24**
Acyl-carrier-protein	Fatty acid and phospholipid metabolism	EF2884	**−**0,16	**−3,18**	**−2,84**
3-oxoacyl-(acyl-carrier-protein) synthase III	Fatty acid and phospholipid metabolism	EF2885	**−**0,39	**−3,68**	**−2,08**
Hypothetical protein	Hypothetical protein	EF2547	**−1,42**	**3,11**	**1,17**
Hypothetical protein	Hypothetical protein	EF3287	**−2,1**	**−1,51**	**−2,56**
Heat shock protein GrpE	Protein fate	EF1307	**1,21**	**1,89**	0,07
DnaK protein	Protein fate	EF1308	**1,34**	**2,30**	**−**0,07
Serine proteinase, V8 family	Protein fate	EF1817	**−**0,01	**−1,82**	**−4,43**
Chaperonin, 10 kDa	Protein fate	EF2634	**1,24**	1,05	0,93
Formate/nitrite transporter family protein	Transport and binding proteins	EF0094	**1,35**	**−2,86**	**−3,46**
Potassium-transporting ATPase, subunit B	Transport and binding proteins	EF0568	0,28	0,66	**2,38**
Potassium-transporting ATPase, subunit C	Transport and binding proteins	EF0569	NA	**−**0,21	**1,20**
Amino acid permease family protein	Transport and binding proteins	EF0635	**1,29**	**−4,13**	**−1,80**
Na+/H+ antiporter	Transport and binding proteins	EF0636	**1,63**	**−3,04**	**−1,18**
Glycine betaine/carnitine/choline ABC transporter, permease protein	Transport and binding proteins	EF0862	**−**0,07	**1,11**	**1,23**
Glycine betaine/carnitine/choline ABC transporter, Glycine betaine/carnitine/choline -binding protein	Transport and binding proteins	EF0863	**−**0,11	**1,43**	**1,33**
Glycine betaine/carnitine/choline ABC transporter, permease protein	Transport and binding proteins	EF0864	**−**0,29	**1,37**	**1,34**
Glycine betaine/carnitine/choline transporter, ATP-binding protein	Transport and binding proteins	EF0865	**−**0,17	**1,38**	1,07
Glycine betaine/L-proline ABC transporter,ATP-binding subunit	Transport and binding proteins	EF2641	**−**0,21	**3,42**	**2,92**
Glycine betaine/L-proline ABC transporter, glycine betaine/L-proline-binding/permease protein	Transport and binding proteins	EF2642	0,69	**4,99**	**4,21**
Glyoxylase family protein	Unknown function	EF2214	**−1,22**	**1,20**	**1,83**

Log_2_ ratios of differentially expressed genes discussed in the results and discussion section, sorted by functional category (cellular role). Significant regulation is indicated in bold.

The numbers of differentially expressed genes, grouped by functional classification according to the TIGR comprehensive microbial resource; CMR (http://cmr.tigr.org/tigr-scripts/CMR/CmrHomePage.cgi), are shown in Figure S2. All functional groups were tested for significant enrichment among the differentially transcribed genes by Fisher’s exact test (*P*<0.05; data not shown). A total of four groups came out as significantly enriched: Purine/pyrimidine/nucleoside/nucleotide (*P* = 4.0e**^−^** ^08^), Fatty acid and phospholipid metabolism (*P* = 1.1e**^−^** ^06^), Protein synthesis (*P* = 2.2e**^−^** ^05^) and Energy metabolism (*P* = 0.00056). The enrichment of genes involved in protein synthesis most likely reflects the reduced growth rate induced by the presence of NaCl, and is consistent with the observations in osmotically challenged *Bacillus subtilis* [27]. Log_2_ ratios of all the genes identified as differentially expressed in cells treated with 6.5% NaCl are shown in Table S1. In addition, the ratios of genes which are further discussed below are displayed in Table 2.

### Validation of Microarray Data

**Figure 2:**
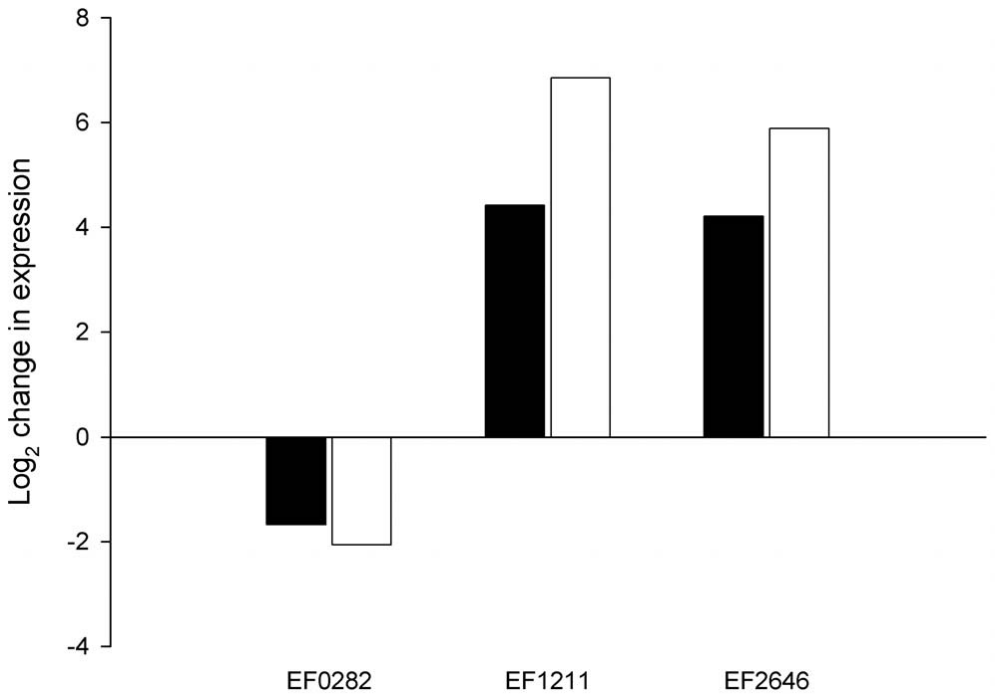
Validation of microarray data by QPCR. The effect of NaCl on the expression of EF0282, EF1211 and EF2642 as quantified by QPCR (□; Pfaffl method), and by microarray (▪).

QPCR was used to validate the microarray analysis. The Pfaffl method [28] was used for relative quantification, and transcriptional data were obtained for the following genes: EF0282, EF1211 and EF2642 at *t_60_*. *dnaB* was used as reference gene. The amplification efficiency varied from 61–102%. The expression levels obtained by QPCR correlated well with the microarray data in terms of direction (Figure 2). The QPCR expression ratios for EF1211 and EF2642 were significantly greater than the microarray values. This may indicate that the change in expression exceeded the dynamic range of the microarray analysis. Indeed, EF1211 and EF2642 were the two genes with the highest positive change in expression at *t_60_* in the microarray data set.

### Conserved Osmotic Stress Response Mechanisms are Activated by the Presence of 6.5% NaCl

**Figure 3:**
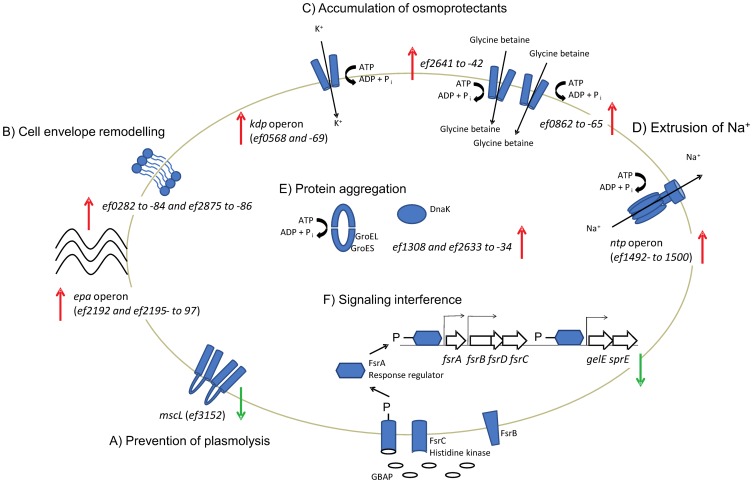
An overview of the main constituents of the *E. faecalis* response to high NaCl concentrations. Among the responses observed was an immediate down-regulation of *mscL* to prevent plasmolysis (A), accompanied by an up-regulation of genes involved in the adjustment of membrane properties (B) and an up-regulation of genes involved in uptake of osmoprotectants (C). The main mechanism for extrusion of excess Na^+^ appeared to be the V-type ATPase encoded by the *ntp* operon (D). An up-regulation of genes coding for the molecular chaperone DnaK and GroEL/ES is indicative of NaCl-induced aggregation and misfolding of proteins (E). Finally, the exposure to NaCl also resulted in a strict repression of the *gelE*-*sprE* operon. Follow-up experiments established that this regulation was due to salt interfering with the receptor-pheromone interaction of the Fsr quorum sensing system (F). Significantly regulated genes are given in parenthesis when only parts of an operon were significantly differentially expressed.

From the transcriptional data it could be inferred that several of the mechanisms activated by the presence of 6.5% NaCl in *E. faecalis* are known from studies of osmotic stress responses in this and other bacteria, and hence expected (Figure 3). Among these conserved mechanisms was a primary response phase including prevention of solute efflux by a 2-fold down-regulation of the large conductance mechanosensitive channel *mscL* (EF3152) with a subsequent accumulation of potassium via the Kdp system. Reportedly, the expression of *kdpFABC* is under control of the KpdDE regulatory system [29], however, the signal transduction cascade of this two component system is not sensitive to elevated osmolarity [30]. On the other hand, adjustments in the membrane phospholipid composition towards higher anionic phospholipid content result in enhanced transcription of *kdpFABC*, as anionic phospholipids regulate the activation of KdpD [31]. NaCl-induced potassium uptake in *E. faecalis* may thus be related to rearrangements of the membrane composition. Experimental evidence of a remodeling of the *E. faecalis* cell envelope upon osmotic upshift is discussed further below.

At high concentrations, monovalent ions such as K^+^ can inhibit various enzymes. A secondary response phase is thus initiated when the intracellular potassium concentration reaches a certain threshold. This phase involves accumulation of compatible solutes, which can accumulate to high concentration to maintain turgor, without affecting cellular functionality [32], [33]. A 2- and 16-fold, respectively, up-regulation of two ABC transporters predicted to be involved in the uptake of glycine betaine (EF0862 to -65 and EF2641 to -42) may suggest that this is the predominant compatible solute taken up by V583 when grown in BHI.

Sodium extrusion by bacteria is generally attributed to antiport of Na^+^ for H^+^ energized by a proton motive force (PMF). In *E. faecalis* however, the ATP-driven V-ATPase represents the primary system for sodium expulsion [34]. Indeed, transcription of the V-ATPase (*ntp* operon; EF1492- to 1500) was significantly up-regulated (6-fold on average) at *t_30_*–*t_60_* in our experiments. The induction of Na^+^-translocating systems emphasizes the importance of retaining cytosolic ion homeostasis. The up-regulation of genes encoding molecular chaperones (DnaK and GroEL/ES) is also in accordance with previous reports [27], and may be indicative of NaCl inducing aggregation and misfolding of proteins in an altered intracellular environment.

### High NaCl Concentration Influences the Expression of Genes Involved in the Cell Envelope Composition

The bacterial cell envelope provides essential protection from the external environment and confers strength and rigidity to counteract the effects of osmotic stress conditions on the cell. Furthermore, osmosensor activity is likely to be mediated through changes in membrane properties. Indeed, differential expression of several gene clusters with membrane/wall-associated functions reflects a major impact of NaCl on the cell envelope. Particularly, genes involved in fatty acid and lipid metabolism were strongly affected. Two gene clusters involved in type II fatty acid synthesis (FAS) (EF0282 to -84 and EF2875 to -86) were significantly repressed (approx. 4-fold) during elevated osmolarity. A similar observation was also made in osmotically challenged *Bacillus subtilis* [27]. The Gram-positive cell envelope is characterized by a cytoplasmic membrane with embedded proteins and lipoteichoic acids (LTA) and cell wall teichoic acids covered by a thick, multilayered peptidoglycan [35]. In addition to peptidoglycan and teichoic acid, a rhamnose-containing polysaccharide has been shown to be the third main constituent of the *E. faecalis* cell wall [35]. Four genes located in the *epa* (enterococcal polysaccharide antigen) gene cluster (EF2192 and EF2195- to 97), including genes that code for rhamnose biosynthesis and glycosyl transferases were 3-4-fold up-regulated at *t_30_* in V583 during treatment with NaCl. Additional genes in the same cluster were also significantly differentially expressed (FDR <0.01), but with log_2_-values <1. Altogether, these results indicate that remodeling of the cell envelope appears to constitute a vital mechanism by which *E. faecalis* counteracts changes in the external osmolarity. This notion is consistent with osmotic stress induced cell envelope modifications in other Gram-positive bacteria [27], [36].

### The Cell Wall Rhamnose Polysaccharide Epa Confers Protection against High NaCl Concentrations

The *epa* gene cluster was originally characterized in *E. faecalis* OG1RF as an antigenic factor during infection in mice [37]. A recent revision of the organization and annotation revealed that *epa* comprises a total of 18 ORFs (*epaA*-*R*) [38]. The gene cluster consists of distinct modules predicted to be responsible for the sequential steps of the polysaccharide biosynthesis process, *i.e.* synthesis of dTDP-rhamnose, glycosyltransferase activity, polymerization and peptidoglycan-linkage, although the exact biochemical functions of the different genes have not been experimentally determined. The Epa polysaccharide has been investigated for its implication in virulence in various animal infection models [39], [40], and has thus been considered as a vital virulence trait of *E. faecalis*. The induction of parts of the *epa* gene cluster during treatment with NaCl suggested that Epa may be involved in the osmotic stress response in *E. faecalis*. To further investigate this notion, a series of experiments providing unequivocal functional genetic evidence for the involvement of the *epa* locus in *E. faecalis* osmoprotection were designed using two different *epa* disruption mutants.

**Figure 4:**
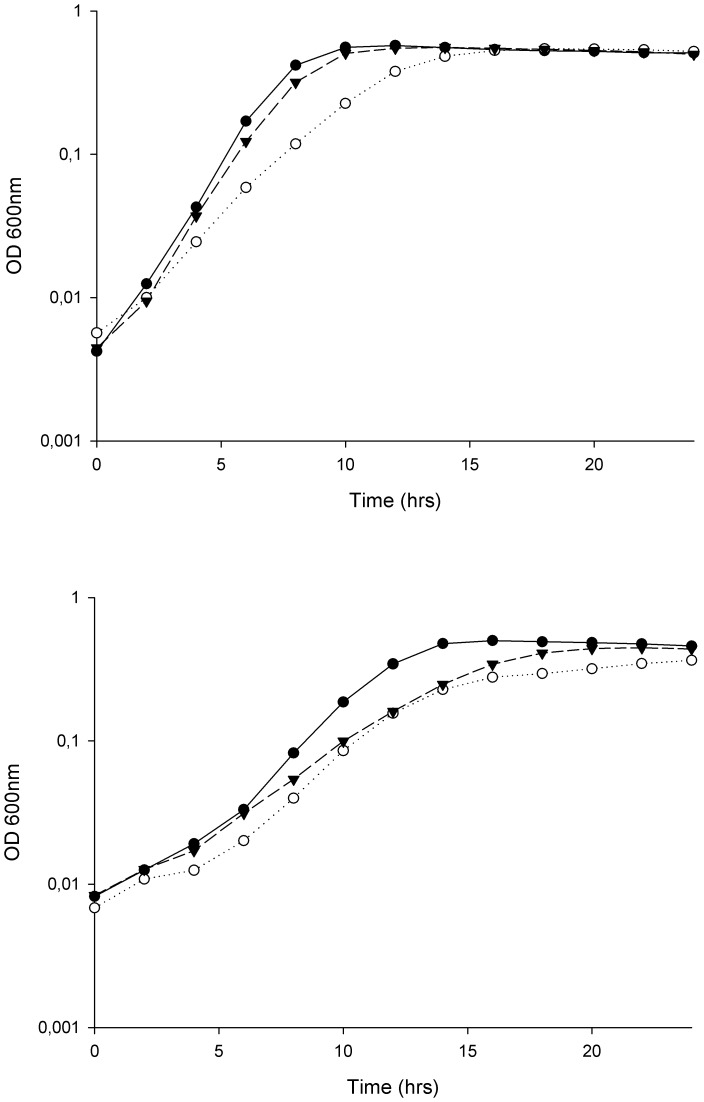
Reduced salt stress resistance in *E. faecalis epaB* (TX5179) and *epaE* (TX5180) mutants. Growth of *E. faecalis* OG1RF (•), TX5179 (○) and TX5180 (▴) treated with 6.5% (top) and 8% NaCl (bottom). All the values are the means from three independent experiments.

**Figure 5:**
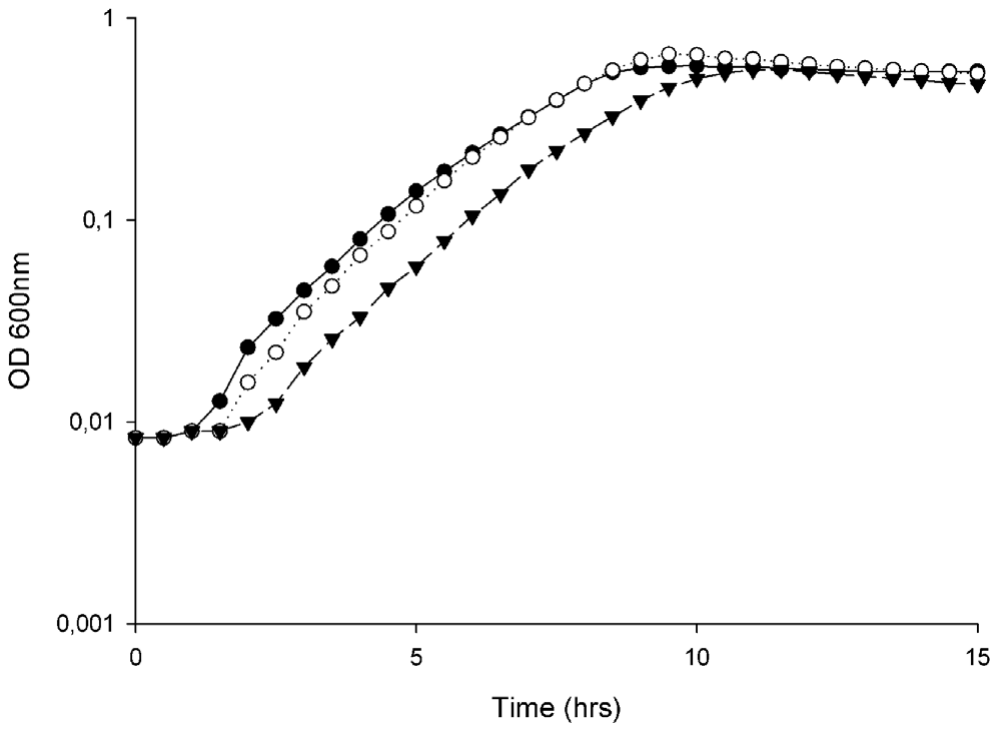
Complementation with *epaBCD* restores wild type salt resistance in *epaB* mutant (TX5179). Growth of *E. faecalis* MS377 (○) and MS381 (▴; mutant carrying empty plasmid) treated with 6.5% NaCl. All the values are the means from three independent experiments. For comparative purposes, the growth of MS383 (•; OG1RF carrying empty plasmid) has been included in the figure.

Xu et al. [41] have previously constructed insertional mutants of OG1RF with disruptions in a glycosyl transferase (*epaB*/*orfde4*) and a glucose-1-phosphate thymidylyltransferase (*epaE*/*orfde6*; TX5179 and TX5180, respectively). Initially, the salt resistance of OG1RF, TX5179 and TX5180 was assessed by investigating their growth in BHI supplemented with 6.5 and 8% NaCl (Figure 4). No significant effects of the mutations were observed in medium without any salt added (result not shown). A moderate but statistically significant reduction in growth rate for TX5180 was observed in the presence of 6.5% NaCl, while the effect on TX5179 was more pronounced (Figure 4; top). In the presence of 8% NaCl, the impairment in growth of the mutants further increased compared to OG1RF (Figure 4; bottom). Moreover, complementation of the Δ*epaB* mutant strain with the *epaBCD* genes cloned in vector pAT28 restored the wild type salt resistance (Figure 5). Unfortunately, despite exhaustive attempts, complementation of the TX5180 mutant using different vectors systems was not achieved. Nevertheless, our results confirmed that the *epa* locus confers increased resistance of *E. faecalis* OG1RF to high NaCl concentrations.

We hypothesized that Epa conferred osmoprotection by either of two mechanisms; *de novo* biosynthesis of rhamnose as a compatible solute, or alternatively that Epa itself, as part of the cell envelope, might act as a protective agent. In order to investigate whether *de novo* biosynthesis and subsequent accumulation of rhamnose served as a mechanism of protection, we tested whether addition of 0.5% rhamnose to the growth medium could alleviate the osmotically induced growth impairment of the different mutants in the presence of 6.5 and 8% NaCl; however, no obvious effect of rhamnose on growth was observed for neither the wild type nor the mutants (results not shown). This observation may be due to the cells not being able to efficiently import rhamnose from the medium under the experimental conditions used. However, if accumulated de novo synthesized rhamnose functioned as an osmoprotector, the salt resistance of the Δ*epaB* mutant strain would be expected to resemble that of the parent strain. Our data thus indicate that the entire Epa biosynthesis pathway, and not only the genes responsible for rhamnose biosynthesis, must be intact in order to confer protection against high NaCl concentrations.

Changes in membrane fluidity have been proposed as a signal for sensors of osmotic stress in other bacteria [42]. It is therefore tempting to speculate that an induction of the parts of the *epa* gene cluster in the present study may be related to a NaCl-induced reduction in membrane fluidity in V583, however this hypothesis was not further tested. Transmission electron microscopy assessment of the cell envelope of *E. faecalis* did not reveal any obvious phenotypic changes in salt stressed cells compared to untreated cells (Figure S3), hence we failed to reproduced the observations reported by Teng and coworkers [38].

### Epa Confers Resistance to Multiple Stress in *E. faecalis*

To evaluate whether the protective effect of Epa was specific to NaCl-induced stress, or if this cell wall polysaccharide also confers resistance to other types of stresses, OG1RF and the *epa* mutants were tested for their sensitivity to biologically relevant stressors. The data revealed that Epa also contributes to resistance to the antimicrobial peptides leucocin C, leucocin A and pediocin PA-1, ethanol, bile acids and the detergent SDS (Table 3). Experiments performed with the complemented mutant confirmed the results for the two leucocins and ethanol, *i.e.* the MIC50 detected for MS377 (complemented mutant) and MS381 (mutant with empty plasmid) were identical to those of V583 and TX5179, respectively. In addition, both the MS377 and MS381 displayed a significant change in resistance to SDS (<0.002%) and sodium deoxycholate (>2.5%) compared to wild type. Notably, the latter experiments were performed in the presence of spectinomycin to ensure the stability of the complementation construct/empty vector. It is possible that the presence of the antibiotic may have affected the physiology of the cell, thus these results should be interpreted with caution. Rigottier-Gois *et al.* [43] recently also demonstrated that *epaB* and *epaN* plays a role in *E. faecalis* resistance to the antibiotics gentamicin and fusidic acid, respectively. Moreover, mutations in *epaA, epaC, epaD* and *epaO* were associated with increased sensitivity to oxidative stress. In line with the data from Rigottier-Gois *et al*. TX5179 did not display altered sensitivity to H_2_O_2_, neither did any of our mutants show altered sensitivity to ampicillin or vancomycin (data not shown). All together, these data indicate that Epa confers resistance to stress that *E. faecalis* is likely to encounter in the human gastrointestinal tract, as well as during infection, thus providing novel clues to the mechanisms by which Epa contributes to enterococcal pathogenicity by rendering *E. faecalis* more stress resistant rather than acting as a classical virulence factor.

**Table 3:** Minimal inhibitory concentrations (MIC).

Stressor	OG1RF	TX5179	TX5180
Leucocin C (AU*/ml)	8	1	1
Leucocin A (AU*/ml)	>2	1	>2
Pediocin PA-1 (AU*/ml)	8	1	8
Ethanol (%)	6	3	6
Sodium dodecyl sulphate (SDS; %)	0.016	0.004	0.004
Sodium taurodeoxycholate (%)	>1	0.08	>1

MIC_50_ was defined as the lowest concentration of the stressor that reduced bacterial growth by >50% in mid exponential growth. Results show average of independent triplicate experiments, no variation was observed. Only the stressors towards which the MIC_50_ of one or more of the mutants deviated from that of the wild type are indicated. AU = arbitrary units.

*1 AU was defined as the bacteriocin concentration necessary for 50% inhibition of growth of *E. faecalis* TX5179 in mid exponential growth (OD_600_ ∼ 0.4 for untreated cells).

### NaCl Interferes with the Fsr Autoregulatory Circuit and Represses gelE-sprE Transcription

One of the most pronounced effects of the presence of 6.5% NaCl in *E. faecalis* was the down regulation of the gelatinase (*gelE;*EF1818) and serine protease (*sprE;* EF1817) at *t_60_*. Both GelE and SprE have been shown to play a role in mammalian and nematode models of enterococcal infection [44]–[46]. The *gelE* and *sprE* genes comprise one transcriptional unit under positive regulation of the Fsr system, via the Fsr binding sequence [47]. The observed down-regulation of *gelE-sprE* is thus suggestive of an interrupted FsrABCD phosphorelay, required for positive regulation of the Fsr-responsive genes.

The *fsr* system is the only autoregulatory three-component system in V583 where signal transduction is mediated by interaction between the histidine kinase FsrC and its cognate peptide pheromone GBAP (gelatinase biosynthesis-activating pheromone) [20]. Bourgogne et al. [48] previously identified a potential Fsr regulon consisting of >450 genes; most of which are probably not directly regulated by Fsr, but whose regulation can be ascribed to indirect effects of the *fsrB* mutation on transcriptional activity. Indeed, the Fsr consensus sequence proposed by Qin et al. [47] was identified in the promoter region of three loci (EF1097, EF1818 and EF1821), whose expression is inferred to be dependent on the phosphorylated FsrA response regulator [48]. Of these putative Fsr-responsive loci, only the *gelE*-*sprE* operon was repressed in *E. faecalis* during exposure to high NaCl concentrations. Transcription of *fsrBDC* was unaffected; an observation which is consistent with the proposed model by Qin et al. [47], who demonstrated that basal transcription of *fsrBDC*, is facilitated by read through from the *fsrA* promoter.

**Figure 6:**
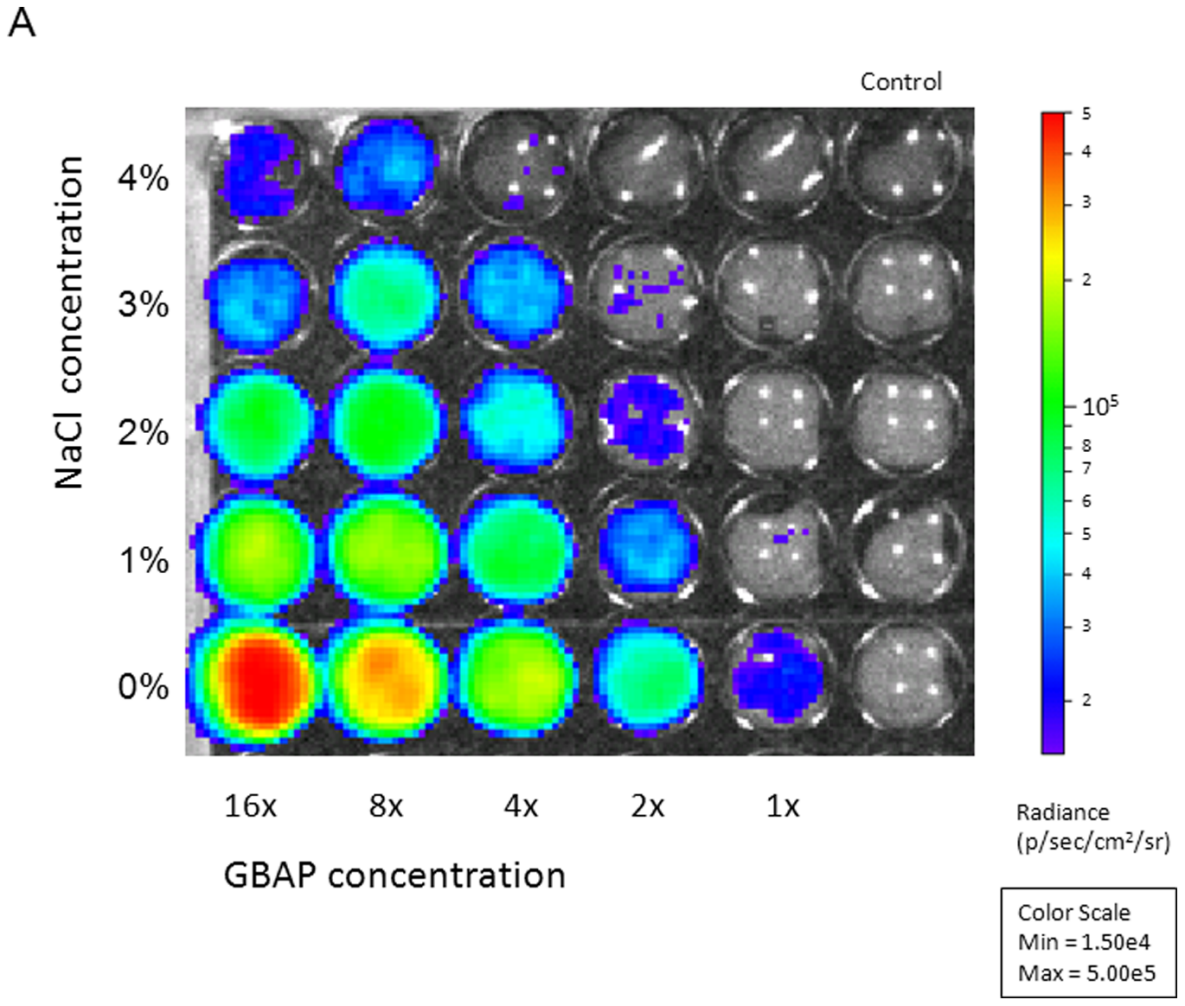
Quantification of *gelE* promoter activity in the GBAP-negative *E. faecalis* OU510. Cells were grown to an optical density at 600∼ 0.2 before the addition of medium containing NaCl and GBAP (gelatinase-biosynthesis activating pheromone). Growth and promoter activity were monitored in replicate plates for 10 h at 37°C. Promoter activity was negatively correlated with NaCl concentration and positively correlated with GBAP concentration. The figure represents a snapshot of the promoter activity 2 hours after the addition of NaCl and GBAP. NaCl concentrations ranged from 0–4%, while there was a 2-fold serial dilution of GBAP from left to right. The images were obtained using an IVIS Lumina II system with an exposure time of 1 min and a binning value of 4. The color bar indicates the signal intensity in p/sec/cm^2^/sr (RLU; relative light units).

**Figure 7:**
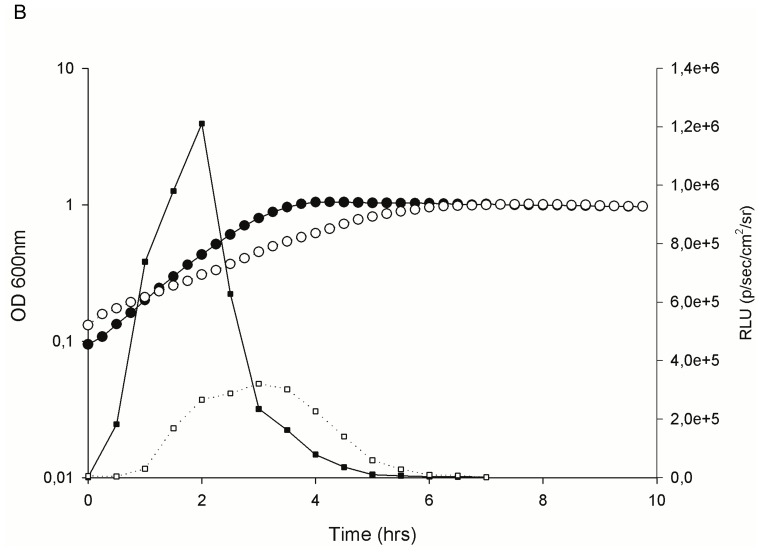
Time course of cell growth and *gelE* promoter activity. Growth of the GBAP-negative *E. faecalis* OU510 with 3% NaCl (○) compared to medium without salt added (•), and promoter activity in response to addition to equal amounts of GBAP measured as RLU (▪ = no NaCl, □ = 3% NaCl). All the values are the means from two independent experiments.

**Figure 8:**
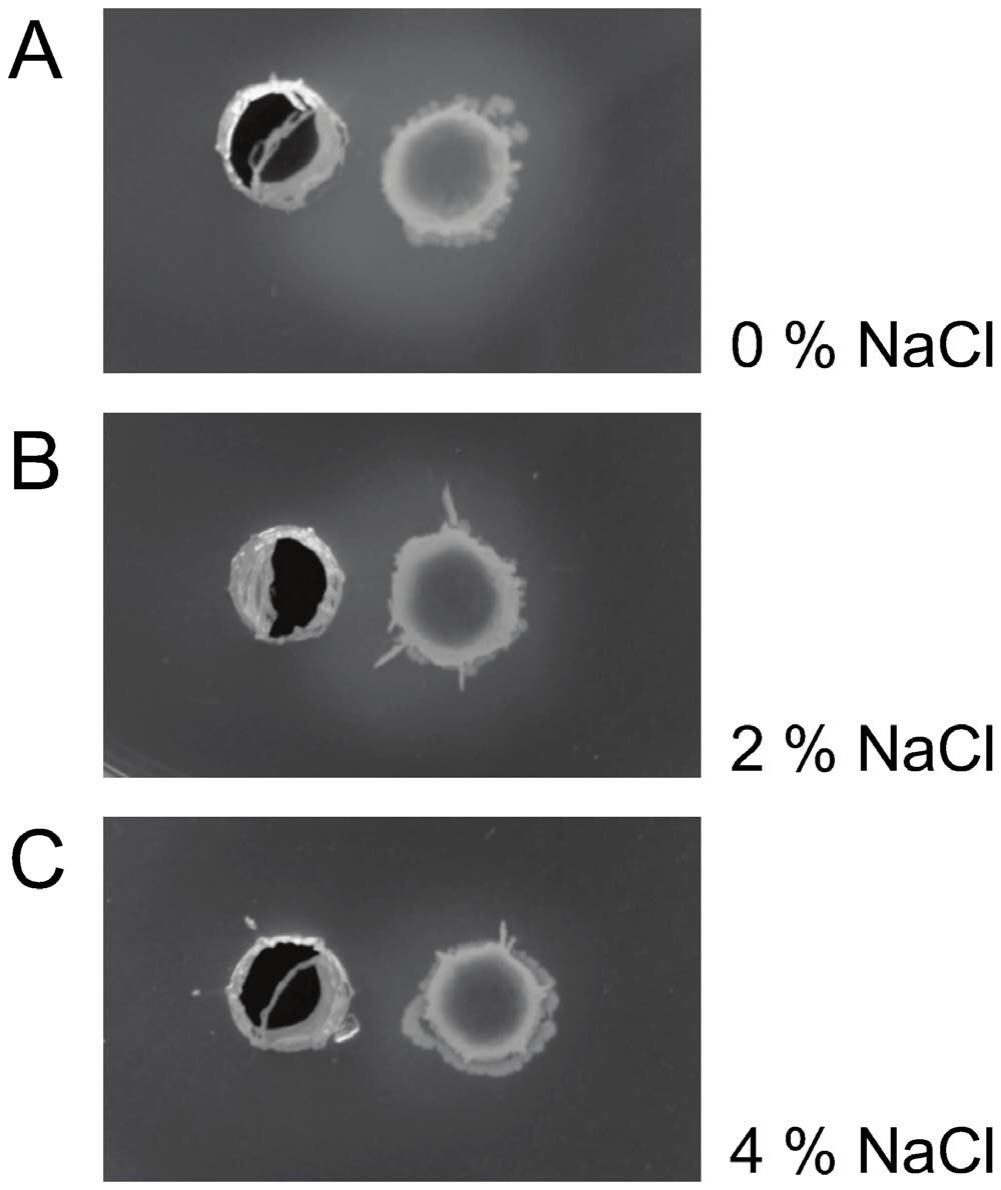
Soft agar plug assays to determine induction of gelatinase activity. The effect of (A) 0%, (B) 2% and (C) 4% NaCl on the ability of equal amount of gelatinase biosynthesis-activating pheromone (GBAP) to induce gelatinase activity in a strain lacking GBAP production was assayed using soft agar plug assays. The right hand pocket was filled in with overnight culture of MS253 diluted 100× in TH soft agar. To the left hand pocket, 1∶1 TH soft agar and sterile filtrated supernatant from overnight culture of MS234, a V583 Δ*gelE* mutant carrying a constitutively expressed version of *fsrD* for overproduction of pheromone was added. A gelatinase negative pheromone producer was used to exclude an effect of carryover of active proteinase. Gelatinase activity was seen as turbid zones around the right hand pocket after incubation at 37°C for 24 h and 4°C for 1 h. After 24 h incubation the effect of NaCl in growth is annulled, in accordance with Figure 1, and the differences in zone size is therefore a result of ionic interference with the Fsr pheromone-receptor interaction.

At the molecular level, hydrophobic and ionic interactions mediate pheromone-receptor binding [49]. These interactions are susceptible to environmental conditions, such as temperature fluctuations, shifts in ionic strength and presence of organic solvents [49], [50]. Hence, we suspected that the down-regulation of *gelE* and *sprE* could be attributed to salt interfering with the receptor-pheromone interaction. To evaluate this hypothesis, we employed a bioluminescence reporter system to investigate the ability of supernatant from a pheromone overproducing *E. faecalis* isolate to induce *fsrB* and *gelE* promoter activity in the non-producing strain *E. faecalis* OU510 in the presence of various concentrations of salt. Promoter activity was negatively correlated with NaCl concentration and positively correlated with pheromone concentration (Figure 6 and 7). The negative correlation between salt concentration and gelatinase activity was also confirmed by soft agar plug assays (Figure 8). Our data indicate that the presence of 3% NaCl resulted in a 4× reduced induction of *gelE* promoter activity measured as RLU, compared to medium with no NaCl added at the same OD_600_ (Figure 7). The corresponding values for 2 and 4% NaCl were approx. 2.5 and 5.3, respectively (results not shown). A slight delay was observed for P*_gelE_* in 3% NaCl, however both cultures peaked in promoter activity at OD_600_ ∼0.45 (Figure 7), indicating that the impaired bioluminescence counts were not a product of the growth retardation, thus supporting our hypothesis. The reduced *gelE* promoter activity caused by the presence of NaCl was also reflected as an altered phenotype, where the presence of 4% NaCl resulted in a severely impaired gelatinase activity (Figure 8). The promoter activity induced by equal amounts of pheromone was ∼8× lower for P*_fsrB_* than for P*_gelE_* in medium with no NaCl added (results not shown), suggesting that FsrA may have a lower affinity for the P*_fsrB_* than to P*_gelE_*. The latter values are consistent with the data obtained by Qin et al. [47] using a β- galactosidase activity assay. Differences in promoter affinity may in turn explain why *gelE*-*sprE* was the only target of the Fsr regulon which displayed detectable differential transcription during the course of this study. On the other hand, more recent data obtained by Del Papa and Perego [51] using electrophoretic mobility shift – and DNAse protection assays do not support differential affinity by FsrA, and our observations may thus also be a results of a more general differential strength of the two promoters, possibly due to differences in the –35 sequence and differences in the distance between the –10 and −35 regions of the promoters.

Notably, GelE activity is key to AtlA ‘fratricide’ mediated release of extracellular DNA (eDNA) and biofilm formation by *E. faecalis* [52]. Moreover, biofilm formation by *E. faecalis* is impeded in the presence of 3% NaCl [53]. NaCl mediated interference of the *fsr* autoregulatory circuit and repression of downstream target *gelE-sprE* genes, may thus prevent activation of AtlA and subsequent release of eDNA. Consequently, our results provide a plausible molecular explanation to the biofilm inhibitory activity of NaCl and demonstrate that biofilm formation by *E. faecalis* could be prevented by targeted interference of the *fsr* autoregulatory circuit. A small number of *fsr* quorum sensing inhibitors have already been identified: In addition to the naturally produced compounds siamycin I [54] and ambuic acid [55], which inhibit the GBAP signal transduction via FsrC-FsrA and GBAP biosynthesis, respectively, an artificial GBAP antagonist was also recently designed [56].

### Conclusion

The transcriptional response of *E. faecalis* to 6.5% NaCl suggests that this organism responds to elevated osmolarity by uptake of compatible solutes and alterations in cell envelope composition. Up-regulation of two gene clusters predicted to encode uptake systems for glycine betaine indicates that this is the predominant compatible solute taken up by V583 when grown in BHI. Growth experiments with *epa* deficient mutants confirmed a role of the enterococcal polysaccharide antigen in *E. faecalis* OG1RF osmoprotection, and led to the elucidation of a more general function of Epa in the *E. faecalis* OG1RF response to several biologically relevant stressors unveiling new insight onto the role of Epa in the ability of *E. faecalis* to cause infection. The *epa* locus is highly conserved among enterococci [57], and Epa constitutes an intrinsic part of the cell envelope and contributes to the robust nature of *E. faecalis*. Moreover, rhamnopolysaccharides are a conserved feature among Gram-positive nosocomial pathogens, including *Listeria* and streptococci (Figure S4). Thus it is highly possible that such cell wall polysaccharides might confer resistance to environmental stressors in a similar manner. Furthermore, the fact that NaCl interference resulted in stringent repression of the *fsr* quorum sensing system, demonstrates that the GBAP pheromone signaling constitutes a potent means for attenuation of GelE and SprE virulence factors and prevention of biofilm development.

## Supporting Information

Figure S1 **The distribution of differentially expressed genes in** ***E. faecalis*** **V583 during growth in NaCl.** Venn diagram showing the number of unique and commonly regulated genes between the time points in the time course experiment.
(TIF)

Figure S2 **Functional classification of differentially expressed genes.** Differentially expressed genes in *E. faecalis* V583 during treatment with NaCl, grouped by functional classification according to the TIGR comprehensive microbial resource; CMR (http://cmr.tigr.org/tigr-scripts/CMR/CmrHomePage.cgi). Black bars refer to induced genes, and grey bars refer to repressed genes. Numbers in parenthesis represent the percentages of the total number of genes within each functional class in the genome. Genes that were both up- and down-regulated during the time course are counted twice.
(TIF)

Figure S3 **The effect of NaCl on cell morphology.** Transmission electron micrographs of *E. faecalis* strains V583 (A and D), OG1RF (B and E) and TX5179 (C and F) grown either in the absence (A–C) of in the presence (D–F) of 6.5% NaCl.
(TIF)

Figure S4 **Conserved rhamnopolysaccharide biosynthesis loci present in Gram-positive nosocomial pathogens compared to the** ***epa*** **cluster in** ***E. faecalis*** **.** Genes are indicated by arrows and colored according to gene function, as indicated. Comparisons were done by tBLASTx using Easyfig 1.2.1.
(TIF)

Table S1 **Primers used in this study.** A list of primer sequences that have been used for PCR, QPCR and cloning.
(DOCX)

Table S2 **Differentially expressed genes.** Log_2_ ratios of genes who were differentially expressed at one or more of the three time points at which the effect of NaCl treatment was studied, sorted by functional category (cellular role). Significant regulation is indicated in bold.
(DOCX)
