# Supplementary figures and images for: Transcriptomic and Functional Analysis of NaCl-Induced Stress in Enterococcus faecalis
Source: PLoS One. 2014 Apr 22;9(4):e94571. doi: 10.1371/journal.pone.0094571 (PMC3995695; doi:10.1371/journal.pone.0094571)

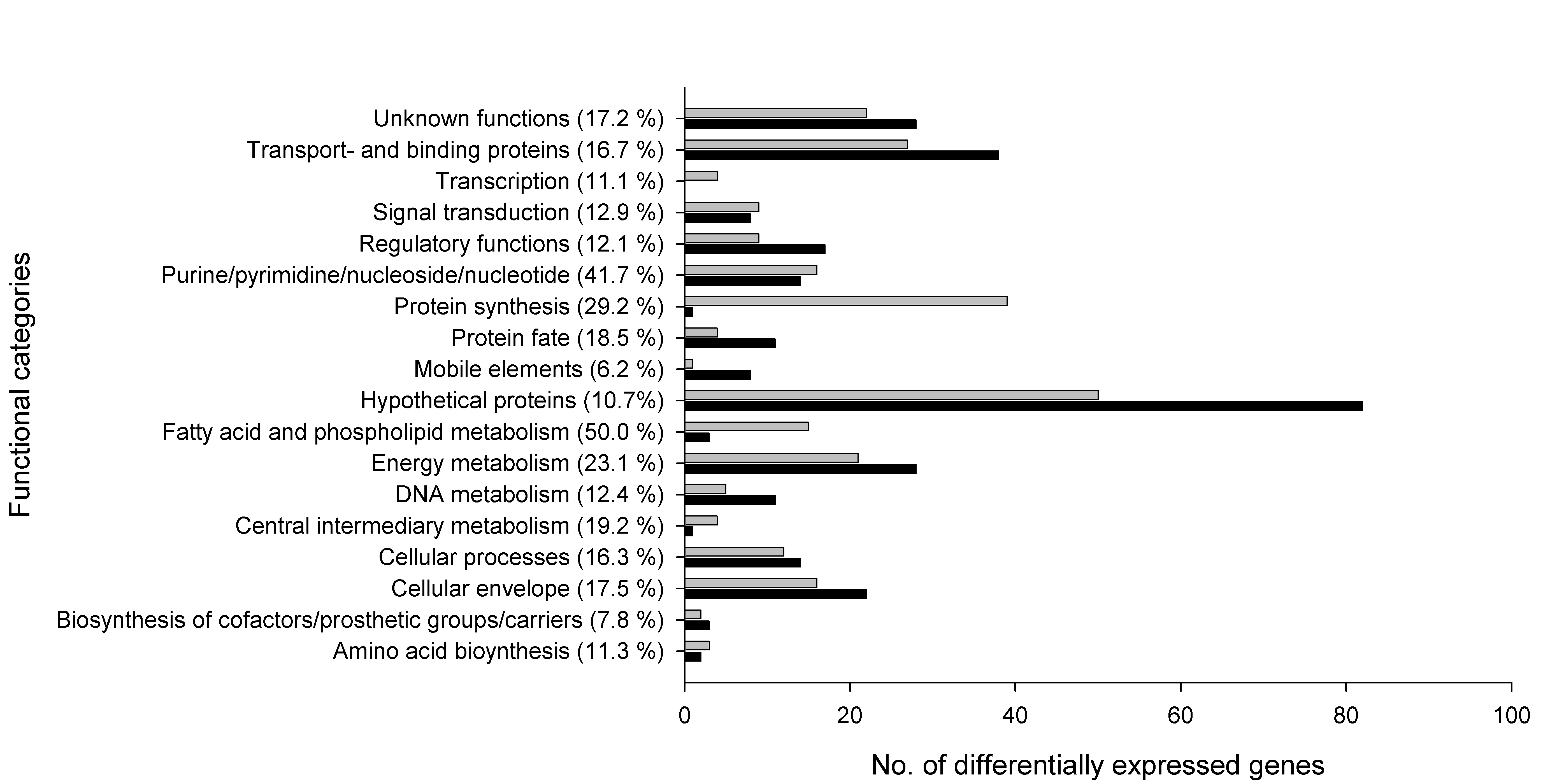

Supplement: Figure S2 — Functional classification of differentially expressed genes. Differentially expressed genes in E. faecalis V583 during treatment with NaCl, grouped by functional classification according to the TIGR comprehensive microbial resource; CMR (http://cmr.tigr.org/tigr-scripts/CMR/CmrHomePage.cgi). Black bars refer to induced genes, and grey bars refer to repressed genes. Numbers in parenthesis represent the percentages of the total number of genes within each functional class in the genome. Genes that were both up- and down-regulated during the time course are counted twice. (TIF) [file pone.0094571.s002.tif]

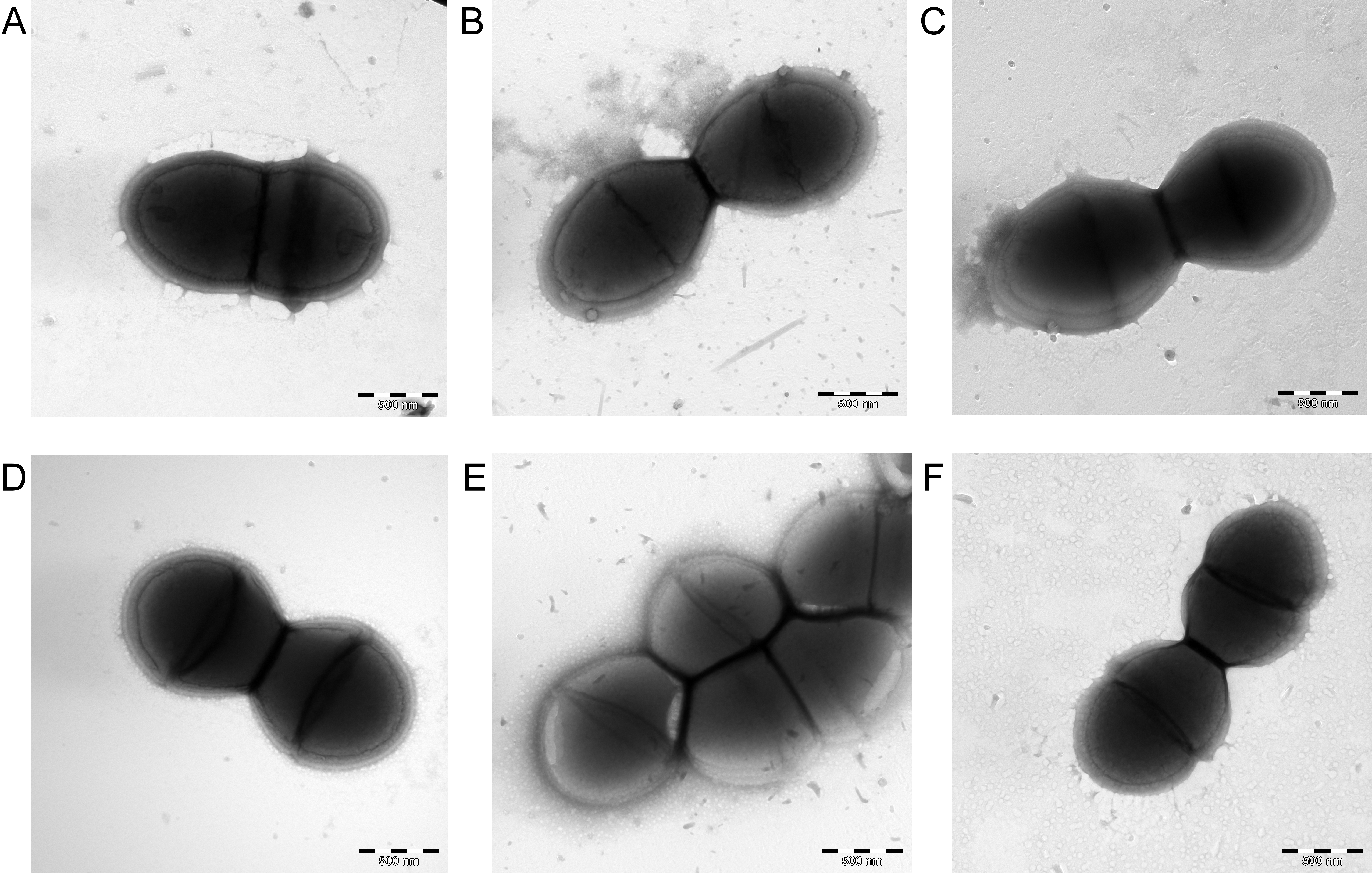

Supplement: Figure S3 — The effect of NaCl on cell morphology. Transmission electron micrographs of E. faecalis strains V583 (A and D), OG1RF (B and E) and TX5179 (C and F) grown either in the absence (A–C) of in the presence (D–F) of 6.5% NaCl. (TIF) [file pone.0094571.s003.tif]

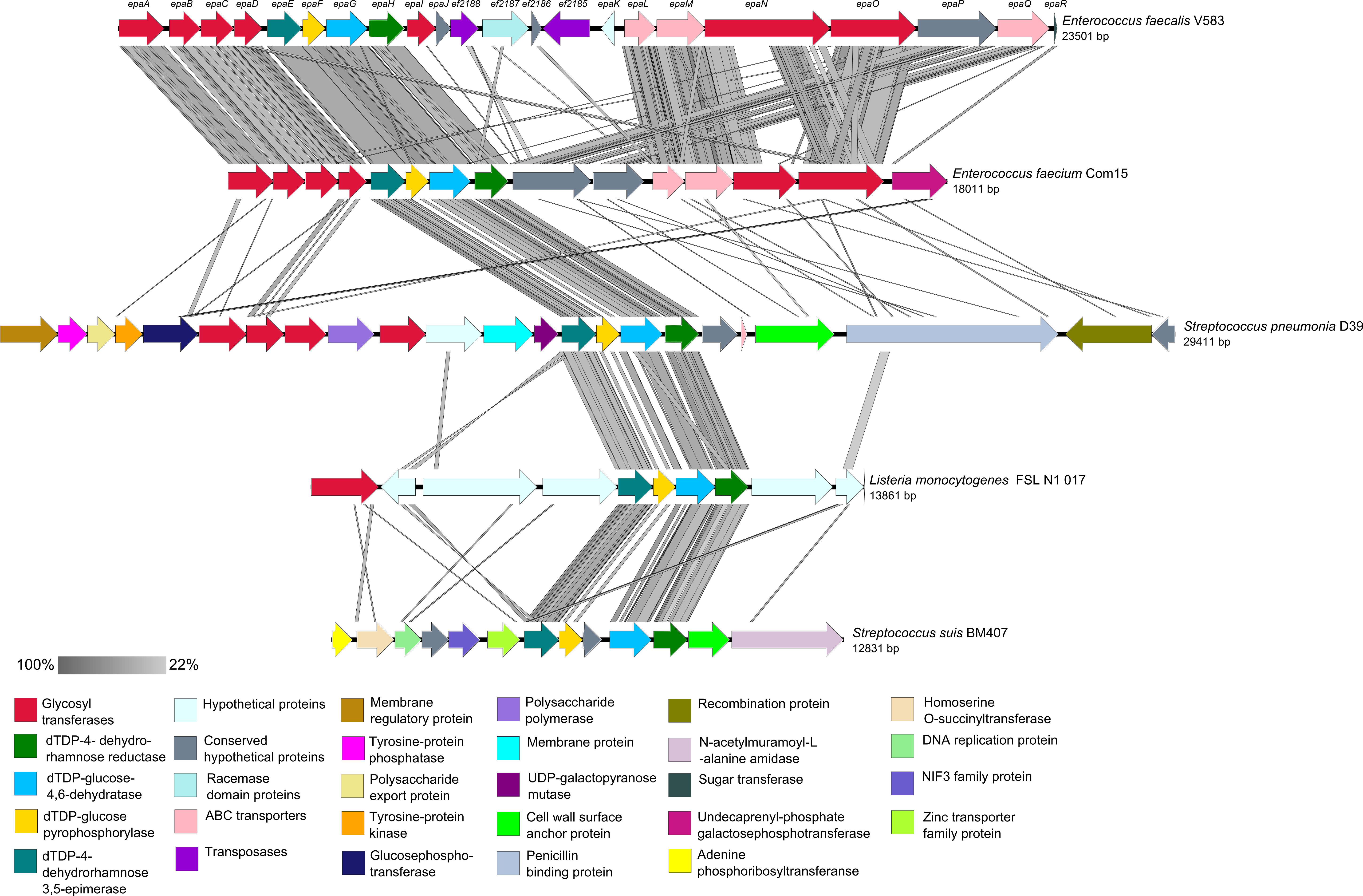

Supplement: Figure S4 — Conserved rhamnopolysaccharide biosynthesis loci present in Gram-positive nosocomial pathogens compared to the epa cluster in E. faecalis . Genes are indicated by arrows and colored according to gene function, as indicated. Comparisons were done by tBLASTx using Easyfig 1.2.1. (TIF) [file pone.0094571.s004.tif]
