# Supplementary material for: Transcriptomic and Functional Analysis of NaCl-Induced Stress in Enterococcus faecalis
Source: PLoS One. 2014 Apr 22;9(4):e94571. doi: 10.1371/journal.pone.0094571 (PMC3995695; doi:10.1371/journal.pone.0094571)
Supplement: Table S1 — Primers used in this study. A list of primer sequences that have been used for PCR, QPCR and cloning. (DOCX) [file pone.0094571.s005.docx]

| **Target gene/**  **primer name** | **Primer sequences (5' → 3')** | **Amplicon size (bp)** | **Application** | **Reference** |
| --- | --- | --- | --- | --- |
|  |  |  |  |  |
| *ef0282* | F. TGA TGG TTT CCT ATT AGC ACA AG | 136 | QPCR | This study |
|  | R: GTT AGG AAT CGC ACG TTC GG |  |  |  |
| *ef1211* | F: AGT GAG CCG GAT GTA TTT GC | 101 | QPCR | [[1](#_ENREF_1)] |
|  | R: TGT TTA CGA GCA TTC GTT GC |  |  |  |
| *ef2642* | F: GTG CTG ATC GTG CTA TTA ACG | 182 | QPCR | This study |
|  | R: AGT GGC ACA CCA ATG ATA ATG G |  |  |  |
| *dnaB* | F: TAG AAA TGG GGG CAG AAT CA | 143 | QPCR | [[2](#_ENREF_2)] |
|  | R: ATT CGC ACG GGA CAA ACT AC |  |  |  |
| *ef2181* | F:ACA CCA AAT CAG GCC AGA AG | 499 | PCR | This study |
|  | R: GGC GCT AAT TCA TCA TCG TT |  |  |  |
| *ef2182* | F:AGG CGA GAT GAT TGG TTT TG | 489 | PCR | This study |
|  | R: TCA CAA AAA CGA CGA ATG GA |  |  |  |
| *ef2183* | F: TAG GAA TTG TCT GGG CGT TT | 497 | PCR | This study |
|  | R:CAT TCC AGT TGG TTG CCA TA |  |  |  |
| *ef2184* | F: TCT GTC TTC TGC TGG TCT GG | 305 | PCR | This study |
|  | R: TCA TAA TCA CAA TGC CGA CAA |  |  |  |
| *ef2189* | F: CAA TAA TGT TTT AAT GCG ATT TTC GTG | 302 | PCR | This study |
|  | R: TGC TAC CAA CCG TGT TAT GG |  |  |  |
| *ef2190* | F: TCG ACA GAT GGA ACC AAA CA | 494 | PCR | This study |
|  | R: CCA AGC CGT TCC ATC ATA TT |  |  |  |
| *ef2191* | F: TGA TGC GGA TAG TAC GTT GG | 498 | PCR | This study |
|  | R: GGC GCT TTT TCT GCA ATA AC |  |  |  |
| *ef2192* | F: TGC CGT AAA AAC AAT GTT CG | 503 | PCR | This study |
|  | R: TGC TCA TAG GCA TCA ACA GG |  |  |  |
| *ef2193* | F: GGT GAT CAT CGT GGC TTT TT | 498 | PCR | This study |
|  | R: AAA CGG ATT TTC CGC TTC A |  |  |  |
| *ef2194* | F: TGC CAA TTT ACG ACA AAC CA | 496 | PCR | This study |
|  | R: TCT GAC GGT TTG ATC CCT TT |  |  |  |
| *ef2195* | F: ATT TTG CAT TTG CGC TTA CG | 502 | PCR | This study |
|  | R: CGT TGT TCC TTT AAA CGC TGA |  |  |  |
| *ef2196* | F: TGG CTG ATA CGC CGA ATT AT | 476 | PCR | This study |
|  | R: TTC TTC CGA AAA TCC TTC CA |  |  |  |
| *ef2197* | F: AAA GCC GAA CTG GGT ACT GA | 509 | PCR | This study |
|  | R: AAC CCA CCA ATT TCA ACG AA |  |  |  |
| *efaBpro-F* | F: GCA AGC ATT CTA AGA GAA AG | 2485^A^ | Cloning | This study |
| *efaD-R* | R: GTA AAA GTT TAG CGT TGT TCC |  |  |  |
| *fsrBpro-F* | F: CCA TCC AAC TCG AGT TCT AGA TTT CCA AAA AGA GGT CG | 480 | Cloning | This study |
| *fsrBpro-R* | R:CAT ATC GCC CTC CTC TTC AAG |  |  |  |
| *gelEpro-F* | F: CCA TCC AAC TCG AGG ATA ACG CGA TTG AAA ACA GTG C | 552 | Cloning | This study |
| *gelEpro-R* | R: CAT CAA ACA ATT AAC TCC TTC CCC |  |  |  |
| *fsrD-F* | F: ACT GCA TGA AAT TTG GTA AAA AAA TAA TTA AAA ATG | 162 | Cloning | This study |
| *fsrD-R* | R: ACT GCT CGA GTC ATT TTT CAA TAT TCT TTT TAG GTT TTT C |  |  |  |

^A^ Product of efaBpro-F /efaD-R

**References**

1. Verneuil N, Rince A, Sanguinetti M, Posteraro B, Fadda G, et al. (2005) Contribution of a PerR-like regulator to the oxidative-stress response and virulence of *Enterococcus faecalis*. Microbiology 151: 3997-4004.

2. Opsata M, Nes IF, Holo H (2010) Class IIa bacteriocin resistance in *Enterococcus faecalis* V583: the mannose PTS operon mediates global transcriptional responses. BMC Microbiol 10: 224.
