# Supplementary material for: Transcriptomic and Functional Analysis of NaCl-Induced Stress in Enterococcus faecalis
Source: PLoS One. 2014 Apr 22;9(4):e94571. doi: 10.1371/journal.pone.0094571 (PMC3995695; doi:10.1371/journal.pone.0094571)
Supplement: Table S2 — Differentially expressed genes. Log2 ratios of genes who were differentially expressed at one or more of the three time points at which the effect of NaCl treatment was studied, sorted by functional category (cellular role). Significant regulation is indicated in bold. (DOCX) [file pone.0094571.s006.docx]

| **Gene product** | **Cellular role** | **ORF** | **Log_2_-ratio at:** |  |  |
| --- | --- | --- | --- | --- | --- |
|  |  |  | **T(5)** | **T(30)** | **T(60)** |
| [Cystathionine gamma-synthase/cystathionine beta-lyase](http://www.ncbi.nlm.nih.gov/entrez/viewer.fcgi?db=protein&val=29374929) | Amino acid biosynthesis | EF0290 | **0,90** | -0,21 | 0,85 |
| Aspartate kinase | Amino acid biosynthesis | EF0368 | 0,81 | **1,38** | 0,27 |
| Shikimate 5-dehydrogenase | Amino acid biosynthesis | EF1561 | -0,02 | -0,88 | **-0,80** |
| Prephenate dehydrogenase | Amino acid biosynthesis | EF1565 | 0,42 | **-1,34** | -0,55 |
| Shikimate kinase | Amino acid biosynthesis | EF1567 | 0,31 | **-1,29** | -0,21 |
| Prephenate dehydratase | Amino acid biosynthesis | EF1568 | 0,12 | **-1,22** | -0,38 |
| Cysteine synthase A | Amino acid biosynthesis | EF1584 | **-0,92** | -0,93 | **1,43** |
| 3-dehydroquinate dehydratase, type I | Amino acid biosynthesis | EF1731 | -0,27 | 1,00 | **0,95** |
| Serine hydroxymethyltransferase | Amino acid biosynthesis | EF2550 | 0,01 | **-1,00** | 0,00 |
| 4-diphosphocytidyl-2C-methyl-D-erythritol kinase | Biosynthesis of cofactors, prosthetic groups, and carriers | EF0051 | 0,72 | **2,93** | 0,05 |
| Naphthoate synthase | Biosynthesis of cofactors, prosthetic groups, and carriers | EF0445 | -0,45 | -0,45 | **1,09** |
| 2-dehydropantoate 2-reductase, putative | Biosynthesis of cofactors, prosthetic groups, and carriers | EF0517 | 0,64 | -0,73 | **-0,97** |
| Hydroxymethylglutaryl-CoA synthase | Biosynthesis of cofactors, prosthetic groups, and carriers | EF1363 | -0,25 | -0,69 | **-0,80** |
| Aspartate α-decarboxylase | Biosynthesis of cofactors, prosthetic groups, and carriers | EF1858 | -0,11 | -1,02 | **-1,40** |
| Ferrochelatase | Biosynthesis of cofactors, prosthetic groups, and carriers | EF1989 | 0,38 | 0,77 | **0,68** |
| HemK protein | Biosynthesis of cofactors, prosthetic groups, and carriers | EF2553 | 0,27 | **-1,91** | -0,76 |
| Cobyric acid synthase, putative | Biosynthesis of cofactors, prosthetic groups, and carriers | EF2586 | -0,06 | 0,22 | **0,68** |
| 1,4-dihydroxy-2-naphthoate octaprenyltransferase, putative | Biosynthesis of cofactors, prosthetic groups, and carriers | EF3254 | **1,38** | -0,34 | **-**0,94 |
| Membrane protein, putative | Cell envelope | EF0032 | -0,13 | **2,27** | **0,80** |
| Lipoprotein, putative | Cell envelope | EF0071 | 0,05 | 1,32 | **1,45** |
| Basic membrane protein familiy | Cell envelope | EF0176 | 0,28 | **-1,32** | -0,26 |
| Basic membrane protein familiy | Cell envelope | EF0177 | 0,41 | **-1,19** | **-**0,53 |
| Membrane protein, putative | Cell envelope | EF0235 | 1,03 | **1,11** | 0,46 |
| Lipoprotein, putative | Cell envelope | EF0304 | 0,23 | -0,62 | **-1,34** |
| Endolysin, putative | Cell envelope | EF0355 | -0,52 | **-**1,08 | **-1,12** |
| Membrane protein, putative | Cell envelope | EF0516 | 0,59 | **-1,58** | **-1,60** |
| Polysaccharide biosynthesis family protein | Cell envelope | EF0559 | 0,07 | **2,15** | 0,28 |
| Membrane protein, putative | Cell envelope | EF0617 | **1,12** | 1,29 | 0,22 |
| NAD-dependent epimerase/dehydratase family protein | Cell envelope | EF0646 | 0,59 | **2,42** | 0,21 |
| Membrane protein, putative | Cell envelope | EF0673 | -0,81 | 0,92 | **1,16** |
| Membrane protein, putative | Cell envelope | EF0809 | **-1,40** | 0,87 | **2,19** |
| Membrane protein, putative | Cell envelope | EF0860 | 0,24 | **-2,45** | -0,68 |
| [Phospho-N-acetylmuramoyl-pentapeptide-transferase](http://www.ncbi.nlm.nih.gov/entrez/viewer.fcgi?db=protein&val=29375574) | Cell envelope | EF0992 | **0,66** | 0,19 | -0,53 |
| [UDP-N-acetylmuramoyl-L-alanyl-D-glutamate synthetase](http://www.ncbi.nlm.nih.gov/entrez/viewer.fcgi?db=protein&val=29375575) | Cell envelope | EF0993 | **1,05** | -0,05 | -0,17 |
| Lipoprotein, putative | Cell envelope | EF1677 | -0,92 | **1,87** | **2,84** |
| Lipoprotein, putative | Cell envelope | EF1796 | **-1,00** | 0,77 | **1,77** |
| Coccolysin | Cell envelope | EF1818 | -0,40 | **-2,68** | **-4,55** |
| [UDP-N-acetylmuramate--L-alanine ligase](http://www.ncbi.nlm.nih.gov/entrez/viewer.fcgi?db=protein&val=29376436) | Cell envelope | EF1908 | 0,14 | **-1,61** | -0,13 |
| Endocarditis specific antigen | Cell envelope | EF2076 | -0,29 | **2,54** | 0,00 |
| Lipoprotein, putative | Cell envelope | EF2144 | -0,25 | -0,82 | **-0,79** |
| Membrane protein, putative | Cell envelope | EF2169 | **0,80** | **-1,94** | -0,01 |
| Membrane protein, putative | Cell envelope | EF2178 | 0,37 | **-1,49** | -0,65 |
| dTDP-glucose 4,6-dehydratase | Cell envelope | EF2192 | 0,42 | **1,11** | 0,37 |
| Glucose-1-phosphate thymidylyltransferase | Cell envelope | EF2194 | 0,30 | **0,98** | 0,03 |
| Glycosyl transferase, group 2 family protein | Cell envelope | EF2195 | **0,69** | **1,60** | 0,44 |
| Glycosyl transferase, group 2 family protein | Cell envelope | EF2196 | 0,71 | **1,72** | 0,25 |
| Glycosyl transferase, group 2 family protein | Cell envelope | EF2197 | 0,59 | **1,90** | 0,79 |
| UDP-galactopyranose mutase | Cell envelope | EF2487 | 0,69 | **-1,12** | **-1,30** |
| Glycosyl transferase, group 2 family protein | Cell envelope | EF2491 | **1,27** | -0,54 | **-1,14** |
| Glycosyl transferase, group 2 family protein | Cell envelope | EF2492 | **0,78** | 0,15 | **-1,06** |
| Lipoprotein, putative | Cell envelope | EF2512 | -0,57 | **1,58** | 0,06 |
| FemAB family protein | Cell envelope | EF2658 | 0,28 | -0,60 | **-0,89** |
| Membrane protein, putative | Cell envelope | EF2708 | **-1,43** | 0,40 | 0,61 |
| [Cell wall surface anchor family protein](http://www.ncbi.nlm.nih.gov/entrez/viewer.fcgi?db=protein&val=29377193) | Cell envelope | EF2713 | **-0,94** | 0,16 | -0,75 |
| DltD protein | Cell envelope | EF2746 | 0,45 | 1,13 | **-1,56** |
| Penicillin-binding protein 2B | Cell envelope | EF2857 | **-1,01** | **1,89** | 0,54 |
| Glycosyl transferase, group 1 family protein | Cell envelope | EF2891 | **1,08** | 0,40 | 0,24 |
| Glycosyl transferase, group 2 family protein | Cell envelope | EF2908 | -0,41 | **1,16** | 0,50 |
| [Rod shape-determining protein](http://www.ncbi.nlm.nih.gov/entrez/viewer.fcgi?db=protein&val=29377519)  MreD | Cell envelope | EF3061 | -0,53 | **1,45** | -0,01 |
| [Rod shape-determining protein](http://www.ncbi.nlm.nih.gov/entrez/viewer.fcgi?db=protein&val=29377519)  MreC | Cell envelope | EF3062 | **-0,68** | **0,94** | 0,25 |
| Lipoprotein, YaeC family | Cell envelope | EF3198 | **-1,99** | **-1,56** | **0,98** |
| Thiamin biosynthesis lipoprotein ApbE, putative | Cell envelope | EF3255 | **1,30** | -0,39 | **-0,79** |
| Pheromone cAD1 precursor lipoprotein | Cell envelope | EF3256 | **1,36** | 0,00 | **-1,28** |
| Gls24 protein | Cellular processes | EF0079 | -1,02 | -0,48 | **1,91** |
| Gls24 protein | Cellular processes | EF0080 | -0,92 | -0,25 | **3,21** |
| Regulatory protein pfoR, putative | Cellular processes | EF0097 | **1,65** | 0,07 | **-1,76** |
| Superoxide dismutase, Mn | Cellular processes | EF0463 | **-2,64** | 0,22 | 1,17 |
| [Low temperature requirement C protein, putative](http://www.ncbi.nlm.nih.gov/entrez/viewer.fcgi?db=protein&val=29375240) | Cellular processes | EF0639 | -0,70 | 0,59 | **1,05** |
| Rhodanese family protein | Cellular processes | EF0748 | **-1,78** | **-0,99** | 0,30 |
| [Cold shock domain-contain protein](http://www.ncbi.nlm.nih.gov/entrez/viewer.fcgi?db=protein&val=29375372) | Cellular processes | EF0781 | **-3,13** | **-1,33** | -0,20 |
| Drug resistance transporter, EmrB/QacA family protein | Cellular processes | EF0785 | **-0,94** | **-1,77** | -0,43 |
| Drug resistance protein, putative | Cellular processes | EF1042 | **-**0,82 | 0,16 | **0,75** |
| Multidrug resistance protein, putative | Cellular processes | EF1078 | 0,68 | -1,58 | **-1,00** |
| [Cold-shock domain-contain protein](http://www.ncbi.nlm.nih.gov/entrez/viewer.fcgi?db=protein&val=29375934) | Cellular processes | EF1367 | -1,13 | -0,79 | **-1,67** |
| Catalase/peroxidase | Cellular processes | EF1597 | **-1,16** | -0,82 | 0,67 |
| [ATP-dependent protease ATP-binding subunit](http://www.ncbi.nlm.nih.gov/entrez/viewer.fcgi?db=protein&val=29376200) | Cellular processes | EF1646 | -0,07 | 0,65 | **1,02** |
| [Cell division ATP-binding protein FtsE](http://www.ncbi.nlm.nih.gov/entrez/viewer.fcgi?db=protein&val=29376310) | Cellular processes | EF1761 | **0,39** | **1,08** | **0,72** |
| Cold shock protein CspC | Cellular processes | EF1991 | **-3,46** | -0,55 | -0,48 |
| Multidrug resistance protein, putative | Cellular processes | EF2068 | -0,84 | **3,19** | 0,28 |
| [Undecaprenyl pyrophosphate phosphatase](http://www.ncbi.nlm.nih.gov/entrez/viewer.fcgi?db=protein&val=29376933) | Cellular processes | EF2439 | -0,32 | -1,13 | -0,75 |
| [Cell cycle protein FtsW](http://www.ncbi.nlm.nih.gov/entrez/viewer.fcgi?db=protein&val=29376951) | Cellular processes | EF2457 | 0,34 | **1,57** | -0,19 |
| [Cell cycle protein FtsW](http://www.ncbi.nlm.nih.gov/entrez/viewer.fcgi?db=protein&val=29376951) | Cellular processes | EF2502 | -0,52 | **1,70** | -0,08 |
| Adaptor protein | Cellular processes | EF2677 | -0,53 | 0,10 | **1,25** |
| [Alkyl hydroperoxide reductase, C subunit](http://www.ncbi.nlm.nih.gov/entrez/viewer.fcgi?db=protein&val=29377215) | Cellular processes | EF2739 | **1,08** | **1,29** | **1,03** |
| [MarR family transcriptional regulator](http://www.ncbi.nlm.nih.gov/entrez/viewer.fcgi?db=protein&val=29377352) | Cellular processes | EF2886 | -0,19 | **-3,16** | **-2,06** |
| Large conductance mechanosensitive channel protein | Cellular processes | EF3152 | **-1,03** | -0,63 | 0,96 |
| Dps family protein | Cellular processes | EF3233 | 0,00 | **2,96** | **1,96** |
| [Abortive phage resistance protein, putative](http://www.ncbi.nlm.nih.gov/entrez/viewer.fcgi?db=protein&val=29377685) | Cellular processes | EF3241 | 0,64 | **2,16** | 0,58 |
| [Transcriptional regulator](http://www.ncbi.nlm.nih.gov/entrez/viewer.fcgi?db=protein&val=29377726) CtsR | Cellular processes | EF3283 | **-1,30** | 0,42 | **1,42** |
| 6-aminoglycoside N-acetyltransferase | Cellular processes | EFA0061 | 0,11 | **-2,60** | **-2,13** |
| ParA family protein | Cellular processes | EFB0064 | 0,29 | **1,59** | -0,23 |
| [Glucosamine-6-phosphate isomerase](http://www.ncbi.nlm.nih.gov/entrez/viewer.fcgi?db=protein&val=29375097) | Central intermediary metabolism | EF0466 | -0,02 | **1,14** | 0,45 |
| [6-aminohexanoate-cyclic-dimer hydrolase, putative](http://www.ncbi.nlm.nih.gov/entrez/viewer.fcgi?db=protein&val=29375612) | Central intermediary metabolism | EF1033 | **-1,18** | -0,10 | 0,37 |
| Glycerol dehydrogenase, putative | Central intermediary metabolism | EF1358 | **-1,11** | -0,43 | **1,89** |
| [Carbonic anhydrase, putative](http://www.ncbi.nlm.nih.gov/entrez/viewer.fcgi?db=protein&val=29376263) | Central intermediary metabolism | EF1711 | **-1,32** | -0,83 | 0,84 |
| [D-fructose-6-phosphate amidotransferase](http://www.ncbi.nlm.nih.gov/entrez/viewer.fcgi?db=protein&val=29376660) | Central intermediary metabolism | EF2151 | 0,07 | **-2,76** | **0,85** |
| [Excinuclease ABC subunit B](http://www.ncbi.nlm.nih.gov/entrez/viewer.fcgi?db=protein&val=29375355) | DNA metabolism | EF0762 | **-1,14** | -0,17 | -0,04 |
| DNA polymerase I | DNA metabolism | EF0878 | **-0,88** | 0,62 | 0,75 |
| Uracil-DNA glycosylase | DNA metabolism | EF0948 | 0,65 | 0,85 | **0,85** |
| [DNA repair exonuclease family protein](http://www.ncbi.nlm.nih.gov/entrez/viewer.fcgi?db=protein&val=29375555) | DNA metabolism | EF0972 | **-0,79** | **2,60** | -0,20 |
| MutT/nudix family protein | DNA metabolism | EF1587 | -0,25 | **1,84** | 0,17 |
| [Phage integrase family site specific recombinase](http://www.ncbi.nlm.nih.gov/entrez/viewer.fcgi?db=protein&val=29376202) | DNA metabolism | EF1648 | -0,28 | **1,27** | **1,10** |
| DNA topoisomerase I | DNA metabolism | EF1650 | -0,20 | **-2,35** | -0,21 |
| Endonuclease IV | DNA metabolism | EF1736 | **-0,85** | -0,87 | **0,85** |
| [Resolvase family site-specific recombinase](http://www.ncbi.nlm.nih.gov/entrez/viewer.fcgi?db=protein&val=29376786) | DNA metabolism | EF2283 | 0,24 | **1,19** | **-0,98** |
| [TOPRIM domain-containing protein](http://www.ncbi.nlm.nih.gov/entrez/viewer.fcgi?db=protein&val=29376807) | DNA metabolism | EF2305 | 0,12 | **1,72** | -0,27 |
| Exonuclease SbcC | DNA metabolism | EF2689 | 0,25 | **1,68** | 0,46 |
| Exonuclease SbcD | DNA metabolism | EF2690 | 0,00 | **1,31** | 0,32 |
| [A/G-specific adenine glycosylase](http://www.ncbi.nlm.nih.gov/entrez/viewer.fcgi?db=protein&val=29377184) | DNA metabolism | EF2704 | -0,38 | 0,51 | **0,87** |
| [Phage integrase family site specific recombinase](http://www.ncbi.nlm.nih.gov/entrez/viewer.fcgi?db=protein&val=29377323) | DNA metabolism | EF2855 | 0,72 | -1,84 | **-1,91** |
| [Competence/damage-inducible protein CinA](http://www.ncbi.nlm.nih.gov/entrez/viewer.fcgi?db=protein&val=29377622) | DNA metabolism | EF3172 | **-1,10** | -0,05 | **1,04** |
| ImpB/MucB/SamB family protein | DNA metabolism | EFA0078 | NA | **2,67** | 0,09 |
| Single-strand binding protein | DNA metabolism | EFB0043 | 0,99 | **2,00** | -0,45 |
| Site-specific recombinase, resolvase family | DNA metabolism | EFC0009 | **1,40** | **3,82** | 0,26 |
| [L-serine dehydratase, iron-sulfur-dependent, beta subunit](http://www.ncbi.nlm.nih.gov/entrez/viewer.fcgi?db=protein&val=29374749) | Energy metabolism | EF0098 | **1,90** | -0,56 | **-1,78** |
| Arginine deiminase | Energy metabolism | EF0104 | -0,07 | 0,78 | **3,36** |
| [NADH-dependent butanol dehydrogenase, putative](http://www.ncbi.nlm.nih.gov/entrez/viewer.fcgi?db=protein&val=29374840) | Energy metabolism | EF0194 | -0,39 | 0,21 | **2,31** |
| [Ribose-5-phosphate isomerase A](http://www.ncbi.nlm.nih.gov/entrez/viewer.fcgi?db=protein&val=29374843) | Energy metabolism | EF0197 | 0,35 | -0,10 | **-1,08** |
| Maltose O-acetyltransferase, putative | Energy metabolism | EF0250 | 0,04 | 0,47 | **0,81** |
| [1-phosphofructokinase](http://www.ncbi.nlm.nih.gov/entrez/viewer.fcgi?db=protein&val=29375314) | Energy metabolism | EF0718 | -0,23 | **-1,06** | **0,99** |
| Methylglyoxal synthase | Energy metabolism | EF0939 | **-1,47** | 0,29 | -0,03 |
| Beta- phosphoglucomutase | Energy metabolism | EF0956 | **-1,76** | -0,55 | **2,16** |
| [Glycosy hydrolase family protein](http://www.ncbi.nlm.nih.gov/entrez/viewer.fcgi?db=protein&val=29375540) | Energy metabolism | EF0957 | -1,62 | -0,75 | **1,24** |
| Pyruvate kinase | Energy metabolism | EF1046 | 0,43 | -1,15 | **-0,81** |
| [Iron-sulfur cluster binding protein](http://www.ncbi.nlm.nih.gov/entrez/viewer.fcgi?db=protein&val=29375685) | Energy metabolism | EF1109 | 0,60 | 1,16 | **1,00** |
| La[ctoylglutathione lyase](http://www.ncbi.nlm.nih.gov/entrez/viewer.fcgi?db=protein&val=29375716) | Energy metabolism | EF1140 | **-1,64** | -1,29 | **1,47** |
| NADH peroxidase | Energy metabolism | EF1211 | -0,55 | **2,39** | **4,42** |
| Alpha-acetolactate decarboxylase | Energy metabolism | EF1214 | **-1,52** | **-1,78** | 0,37 |
| [Glycosyl hydrolase family protein](http://www.ncbi.nlm.nih.gov/entrez/viewer.fcgi?db=protein&val=29375816) | Energy metabolism | EF1243 | -0,20 | -0,83 | **0,94** |
| [Thioredoxin reductase](http://www.ncbi.nlm.nih.gov/entrez/viewer.fcgi?db=protein&val=29375905) | Energy metabolism | EF1338 | **-1,02** | **-1,74** | -0,18 |
| [Glucan 1,6-alpha-glucosidase, putative](http://www.ncbi.nlm.nih.gov/entrez/viewer.fcgi?db=protein&val=29375915) | Energy metabolism | EF1348 | -0,88 | -0,31 | **2,28** |
| [Glycosyl hydrolase family protein](http://www.ncbi.nlm.nih.gov/entrez/viewer.fcgi?db=protein&val=29375816) | Energy metabolism | EF1349 | -1,14 | -0,58 | **2,42** |
| [Pyruvate dehydrogenase complex E1 component, alpha subunit](http://www.ncbi.nlm.nih.gov/entrez/viewer.fcgi?db=protein&val=29375920) | Energy metabolism | EF1353 | **-1,54** | -0,59 | **1,44** |
| Pyruvate dehydrogenase complex E1 component, beta subunit | Energy metabolism | EF1354 | **-1,44** | -0,67 | **1,28** |
| Pyruvate dehydrogenase complex E2 component, dihydrolipoamide acetyltransferase | Energy metabolism | EF1355 | **-1,55** | -1,03 | **1,18** |
| Pyruvate dehydrogenase complex E3 component, dihydrolipoamide dehydrogenase | Energy metabolism | EF1356 | **-1,67** | -0,82 | **1,48** |
| V-type ATPase, subunit F | Energy metabolism | EF1492 | -0,41 | **2,83** | **1,33** |
| V-type ATPase, subunit I | Energy metabolism | EF1493 | 0,03 | **2,85** | **1,64** |
| V-type ATPase, subunit K | Energy metabolism | EF1494 | 0,32 | **3,14** | **1,74** |
| V-type ATPase, subunit C | Energy metabolism | EF1496 | 0,21 | **3,21** | **2,05** |
| V-type ATPase, subunit G | Energy metabolism | EF1497 | 0,38 | **2,83** | **1,79** |
| V-type ATPase, subunit A | Energy metabolism | EF1498 | 0,33 | **3,10** | **1,76** |
| V-type ATPase, subunit B | Energy metabolism | EF1499 | 0,46 | **3,35** | **2,52** |
| V-type ATPase, subunit D | Energy metabolism | EF1500 | 0,24 | 1,98 | **1,53** |
| Ferredoxin | Energy metabolism | EF1543 | **-0,96** | -0,54 | 0,33 |
| NADH oxidase | Energy metabolism | EF1586 | -1,81 | **-2,05** | **1,66** |
| Pyruvate formate-lyase activating enzyme | Energy metabolism | EF1612 | **1,09** | -0,47 | -0,22 |
| Glycerol-3-phosphate phosphate (NAD(P)+) | Energy metabolism | EF1747 | 0,11 | **1,31** | 0,44 |
| Glyceraldehyde 3- phosphate dehydrogenases | Energy metabolism | EF1964 | 0,01 | 0,66 | **1,13** |
| Cytochrome d ubiquinol oxidase, subunit I | Energy metabolism | EF2061 | **-0,83** | -0,56 | -0,47 |
| Pyruvate carboxylase | Energy metabolism | EF2456 | 0,37 | **1,51** | 0,26 |
| Fumarate reductase flavoprotein subunit precursor, putative | Energy metabolism | EF2556 | 0,65 | **-1,40** | **-1,78** |
| ATP synthase F1, epsilon subunit | Energy metabolism | EF2607 | 0,19 | **-1,56** | **-0,73** |
| ATP synthase F1, beta subunit | Energy metabolism | EF2608 | 0,34 | **-1,21** | -0,34 |
| ATP synthase F1, gamma subunit | Energy metabolism | EF2609 | 0,20 | **-1,62** | -0,85 |
| ATP synthase F1, alpha subunit | Energy metabolism | EF2610 | 0,27 | **-1,66** | -0,60 |
| ATP synthase F1, delta subunit | Energy metabolism | EF2611 | 0,33 | **-1,55** | -0,43 |
| ATP synthase F0, B subunit | Energy metabolism | EF2612 | 0,24 | **-1,45** | -0,36 |
| ATP synthase F0, C subunit | Energy metabolism | EF2613 | 0,46 | **-1,54** | -0,29 |
| ATP synthase F0, A subunit | Energy metabolism | EF2614 | 0,05 | **-1,53** | -0,12 |
| Sucrose-6-phosphate hydrolase | Energy metabolism | EFA0069 | -0,06 | 0,38 | **1,88** |
| Lipase/acylhydrolase | Fatty acid and phospholipid metabolism | EF0169 | **-1,04** | 0,73 | 1,20 |
| Enoyl -(acyl-carrier-protein) reductase | Fatty acid and phospholipid metabolism | EF0282 | -0,56 | **-2,95** | **-1,67** |
| 3-oxoacyl -(acyl-carrier-protein) reductase | Fatty acid and phospholipid metabolism | EF0283 | -0,17 | **-2,11** | **-2,44** |
| (3R)- -(acyl-carrier-protein) reductase | Fatty acid and phospholipid metabolism | EF0284 | -0,23 | **-1,99** | -1,93 |
| Cardiolipin synthetase, putative | Fatty acid and phospholipid metabolism | EF0631 | 0,21 | -0,28 | **-0,91** |
| Short chain dehydrogenase | Fatty acid and phospholipid metabolism | EF1773 | 0,37 | **1,47** | **1,02** |
| Glycerophosphoryl diester phosphodiesterase family protein | Fatty acid and phospholipid metabolism | EF1904 | -0,43 | **1,90** | -0,14 |
| Acyl carrier protein, putative | Fatty acid and phospholipid metabolism | EF2601 | 0,16 | -0,45 | **-1,09** |
| Acetyl-CoA carboxylase, carboxyl transferase alpha subunit | Fatty acid and phospholipid metabolism | EF2875 | 0,04 | **-2,24** | **-1,49** |
| Acetyl-CoA carboxylase, carboxyl transferase beta subunit | Fatty acid and phospholipid metabolism | EF2876 | -0,03 | **-2,58** | **-2,72** |
| Acetyl-CoA carboxylase, biotin carboxylase | Fatty acid and phospholipid metabolism | EF2877 | -0,10 | **-2,51** | **-2,34** |
| (3R)-hydroxymyristoyl-(acyl-carrier-protein) dehydratase | Fatty acid and phospholipid metabolism | EF2878 | -0,18 | **-2,31** | -1,97 |
| Acetyl-CoA carboxylase, biotin carboxyl carrier protein | Fatty acid and phospholipid metabolism | EF2879 | -0,28 | -2,24 | **-1,80** |
| 3-oxoacyl-(acyl-carrier-protein) synthase II | Fatty acid and phospholipid metabolism | EF2880 | 0,31 | **-1,94** | **-2,03** |
| Malonyl CoA-acyl carrier protein transacylase | Fatty acid and phospholipid metabolism | EF2882 | 0,21 | **-2,47** | **-1,71** |
| Enoyl-(acyl-carrier-protein) reductase II | Fatty acid and phospholipid metabolism | EF2883 | -0,08 | **-3,35** | **-2,24** |
| Acyl-carrier-protein | Fatty acid and phospholipid metabolism | EF2884 | -0,16 | **-3,18** | **-2,84** |
| 3-oxoacyl-(acyl-carrier-protein) synthase III | Fatty acid and phospholipid metabolism | EF2885 | -0,39 | **-3,68** | **-2,08** |
| Lipase, putative | Fatty acid and phospholipid metabolism | EF3191 | **1,05** | 0,64 | 0,35 |
| Hypothetical protein | Hypothetical protein | EF0083 | -0,70 | -0,81 | **-1,73** |
| Hypothetical protein | Hypothetical protein | EF0183 | 0,69 | **1,89** | 0,65 |
| Hypothetical protein | Hypothetical protein | EF0184 | **1,05** | **2,45** | 0,76 |
| Hypothetical protein | Hypothetical protein | EF0248 | 0,18 | -0,48 | **-1,52** |
| Hypothetical protein | Hypothetical protein | EF0288 | -0,59 | **-1,30** | **-1,06** |
| Hypothetical protein | Hypothetical protein | EF0375 | -0,13 | **2,20** | **1,72** |
| Hypothetical protein | Hypothetical protein | EF0478 | 0,07 | **-1,07** | -0,56 |
| Hypothetical protein | Hypothetical protein | EF0610 | **1,21** | 0,23 | -1,30 |
| Hypothetical protein | Hypothetical protein | EF0637 | **-0,92** | 0,52 | **1,42** |
| Hypothetical protein | Hypothetical protein | EF0652 | NA | **1,76** | -0,01 |
| Hypothetical protein | Hypothetical protein | EF0773 | **-1,00** | **-0,89** | -0,48 |
| Hypothetical protein | Hypothetical protein | EF0802 | 0,19 | **3,94** | 0,22 |
| Hypothetical protein | Hypothetical protein | EF0925 | **-0,88** | 0,59 | 0,90 |
| Hypothetical protein | Hypothetical protein | EF0953 | **0,64** | 0,32 | -0,34 |
| Hypothetical protein | Hypothetical protein | EF0959 | **-1,47** | -0,42 | **1,74** |
| Hypothetical protein | Hypothetical protein | EF0963 | -0,20 | 0,61 | **1,12** |
| Hypothetical protein | Hypothetical protein | EF1022 | **-1,41** | -1,09 | **1,22** |
| Hypothetical protein | Hypothetical protein | EF1107 | 0,62 | **1,73** | **1,62** |
| Hypothetical protein | Hypothetical protein | EF1192 | 0,12 | **-**0,89 | **-1,43** |
| Hypothetical protein | Hypothetical protein | EF1248 | **-1,61** | **-2,91** | **-0,89** |
| Hypothetical protein | Hypothetical protein | EF1258 | 0 | **1,28** | 0,18 |
| Hypothetical protein | Hypothetical protein | EF1263 | **0,82** | **1,89** | -0,33 |
| Hypothetical protein | Hypothetical protein | EF1315 | -0,08 | -0,10 | **-0,79** |
| Hypothetical protein | Hypothetical protein | EF1501 | -0,03 | 1,32 | **1,09** |
| Hypothetical protein | Hypothetical protein | EF1686 | **-0,91** | 0,93 | -0,62 |
| Hypothetical protein | Hypothetical protein | EF1734 | -0,05 | 0,29 | **- 1,03** |
| Hypothetical protein | Hypothetical protein | EF1735 | **-1,10** | -0,33 | 0,64 |
| Hypothetical protein | Hypothetical protein | EF1737 | **-1,03** | -0,30 | **1,24** |
| Hypothetical protein | Hypothetical protein | EF1853 | 0,1 | **1,79** | 0,86 |
| Hypothetical protein | Hypothetical protein | EF1925 | 0,26 | 0,49 | **-1,56** |
| Hypothetical protein | Hypothetical protein | EF1933 | **-1,16** | 0,35 | **1,57** |
| Hypothetical protein | Hypothetical protein | EF1936 | -0,56 | **-1,45** | **-1,45** |
| Hypothetical protein | Hypothetical protein | EF1944 | -1,07 | 0,09 | **1,79** |
| Hypothetical protein | Hypothetical protein | EF2019 | 0,23 | 0,14 | **-1,95** |
| Hypothetical protein | Hypothetical protein | EF2308 | -0,22 | **2,08** | 0,66 |
| Hypothetical protein | Hypothetical protein | EF2309 | -0,32 | **1,60** | 0,34 |
| Hypothetical protein | Hypothetical protein | EF2342 | 0,02 | **1,67** | -0,14 |
| Hypothetical protein | Hypothetical protein | EF2405 | 0,18 | -0,79 | **-0,71** |
| Hypothetical protein | Hypothetical protein | EF2427 | -0,05 | **1,38** | -0,05 |
| Hypothetical protein | Hypothetical protein | EF2465 | 0,08 | **2,66** | 0,09 |
| Hypothetical protein | Hypothetical protein | EF2547 | **-1,42** | **3,11** | **1,17** |
| Hypothetical protein | Hypothetical protein | EF2615 | -0,12 | **-1,78** | **-1,21** |
| Hypothetical protein | Hypothetical protein | EF2702 | **-1,36** | 0,46 | 0,98 |
| Hypothetical protein | Hypothetical protein | EF2796 | 0,60 | **2,09** | **2,03** |
| Hypothetical protein | Hypothetical protein | EF2893 | **-0,79** | 1,10 | -0,11 |
| Hypothetical protein | Hypothetical protein | EF2896 | -0,45 | **1,86** | -0,01 |
| Hypothetical protein | Hypothetical protein | EF2950 | 0,28 | **1,89** | 0,23 |
| Hypothetical protein | Hypothetical protein | EF2952 | 0,20 | **1,24** | -0,18 |
| Hypothetical protein | Hypothetical protein | EF2953 | 0,16 | **1,83** | 0,36 |
| Hypothetical protein | Hypothetical protein | EF3039 | -0,22 | 0,94 | **1,13** |
| Hypothetical protein | Hypothetical protein | EF3287 | **-2,1** | **-1,51** | **-2,56** |
| Hypothetical protein | Hypothetical protein | EFA0017 | -0,68 | **1,37** | 0,49 |
| Hypothetical protein | Hypothetical protein | EFA0075 | **1,07** | **4,08** | -0,36 |
| Hypothetical protein | Hypothetical protein | EFC0012 | 0,82 | **2,39** | 0,17 |
| Hypothetical protein | Hypothetical protein | EFC0014 | NA | **2,40** | -0,47 |
| Hypothetical protein | Hypothetical protein | EFC0016 | 0,14 | 0,73 | -0,07 |
| Hypothetical protein | Hypothetical protein | EFC0017 | -0,13 | **2,28** | -1,09 |
| Conserved hypothetical protein | Hypothetical protein - conserved | EF0003 | -0,45 | **2,06** | 0,58 |
| Conserved hypothetical protein | Hypothetical protein - conserved | EF0024 | -0,50 | **-1,96** | -1,17 |
| Conserved hypothetical protein | Hypothetical protein - conserved | EF0050 | -0,68 | **3,29** | -0,73 |
| Conserved hypothetical protein | Hypothetical protein - conserved | EF0077 | **-0,91** | 0,17 | **3,11** |
| Conserved hypothetical protein | Hypothetical protein - conserved | EF0078 | -0,80 | 0,36 | **3,75** |
| Conserved hypothetical protein | Hypothetical protein - conserved | EF0081 | -0,74 | 0,23 | **1,85** |
| Conserved hypothetical protein | Hypothetical protein - conserved | EF0091 | -0,56 | -0,59 | **0,91** |
| Conserved hypothetical protein | Hypothetical protein - conserved | EF0127 | **-0,59** | 0,04 | **-1,18** |
| Conserved hypothetical protein | Hypothetical protein - conserved | EF0170 | -0,36 | 1,10 | **1,21** |
| Conserved hypothetical protein | Hypothetical protein - conserved | EF0241 | **-0,95** | 0,24 | **1,07** |
| Conserved hypothetical protein | Hypothetical protein - conserved | EF0397 | -0,38 | 0,99 | **1,23** |
| Conserved hypothetical protein | Hypothetical protein - conserved | EF0400 | **0,73** | -1,49 | -1,80 |
| Conserved hypothetical protein | Hypothetical protein - conserved | EF0546 | **0,95** | **-0,99** | **-0,51** |
| Conserved hypothetical protein | Hypothetical protein - conserved | EF0667 | **-1,25** | 0,05 | **1,07** |
| Conserved hypothetical protein | Hypothetical protein - conserved | EF0766 | -0,10 | **1,10** | 0,22 |
| Conserved hypothetical protein | Hypothetical protein - conserved | EF0770 | -0,74 | 0,09 | **1,23** |
| Conserved hypothetical protein | Hypothetical protein - conserved | EF0774 | -0,28 | **1,05** | 0,39 |
| Conserved hypothetical protein | Hypothetical protein - conserved | EF0819 | NA | **2,80** | 0,42 |
| Conserved hypothetical protein | Hypothetical protein - conserved | EF0857 | -0,22 | -0,78 | **-1,45** |
| Conserved hypothetical protein TIGR00244 | Hypothetical protein - conserved | EF0881 | -0,33 | **1,12** | 0,42 |
| Conserved hypothetical protein | Hypothetical protein - conserved | EF0906 | 0,44 | **2,03** | **1,67** |
| Conserved hypothetical protein | Hypothetical protein - conserved | EF0908 | 0,43 | **1,87** | 0,64 |
| Conserved hypothetical protein | Hypothetical protein - conserved | EF0937 | 0,83 | **1,98** | 0,77 |
| Conserved hypothetical protein | Hypothetical protein - conserved | EF0940 | 0,03 | **-1,03** | **-2,02** |
| Conserved hypothetical protein | Hypothetical protein - conserved | EF0971 | **-1,00** | -0,51 | -0,12 |
| Conserved hypothetical protein TIGR00242 | Hypothetical protein - conserved | EF0988 | -0,22 | **1,35** | 0,21 |
| Conserved hypothetical protein | Hypothetical protein - conserved | EF1021 | -0,48 | 1,29 | **1,86** |
| Conserved hypothetical protein | Hypothetical protein - conserved | EF1023 | 0,33 | -0,40 | **-1,19** |
| Conserved hypothetical protein | Hypothetical protein - conserved | EF1047 | -0,26 | **-1,83** | -0,70 |
| Conserved hypothetical protein | Hypothetical protein - conserved | EF1145 | -0,60 | **-1,08** | 0,19 |
| Conserved hypothetical protein | Hypothetical protein - conserved | EF1150 | **-0,96** | 0,80 | **1,39** |
| Conserved hypothetical protein | Hypothetical protein - conserved | EF1180 | -1,26 | 0,82 | **2,47** |
| Conserved hypothetical protein | Hypothetical protein - conserved | EF1190 | **-0,85** | **-1,79** | **-1,34** |
| Conserved hypothetical protein | Hypothetical protein - conserved | EF1227 | 0,98 | -0,62 | **-1,08** |
| Conserved hypothetical protein | Hypothetical protein - conserved | EF1247 | **-2,65** | **-2,22** | -0,27 |
| Conserved hypothetical protein | Hypothetical protein - conserved | EF1311 | 0,34 | **1,24** | 0,15 |
| Conserved hypothetical protein | Hypothetical protein - conserved | EF1313 | -0,36 | -0,19 | -0,40 |
| Conserved hypothetical protein | Hypothetical protein - conserved | EF1368 | -0,59 | 1,22 | **2,54** |
| Conserved hypothetical protein | Hypothetical protein - conserved | EF1371 | -0,54 | **1,17** | 0,35 |
| Conserved hypothetical protein | Hypothetical protein - conserved | EF1376 | 0,26 | **-1,16** | 0,24 |
| Conserved hypothetical protein | Hypothetical protein - conserved | EF1419 | 0,27 | **-1,23** | **-0,90** |
| Conserved hypothetical protein | Hypothetical protein - conserved | EF1505 | **-0,98** | -1,03 | 0,25 |
| Conserved hypothetical protein | Hypothetical protein - conserved | EF1518 | **-1,87** | 0,61 | 0,45 |
| Conserved hypothetical protein | Hypothetical protein - conserved | EF1609 | **-0,98** | -0,49 | 0,52 |
| Conserved hypothetical protein | Hypothetical protein - conserved | EF1664 | -0,09 | 1,07 | **1,10** |
| Conserved hypothetical protein | Hypothetical protein - conserved | EF1702 | -0,21 | **2,15** | 0,55 |
| Conserved hypothetical protein | Hypothetical protein - conserved | EF1738 | **-1,12** | -0,05 | **2,41** |
| Conserved hypothetical protein | Hypothetical protein - conserved | EF1745 | -0,23 | **1,24** | **0,84** |
| Conserved hypothetical protein | Hypothetical protein - conserved | EF1792 | 0,14 | **1,65** | 0,76 |
| Conserved hypothetical protein | Hypothetical protein - conserved | EF1794 | -0,81 | 1,64 | **2,40** |
| Conserved hypothetical protein | Hypothetical protein - conserved | EF1797 | 0,21 | 0,70 | **-0,97** |
| Conserved hypothetical protein | Hypothetical protein - conserved | EF1903 | **-1,16** | **2,19** | 0,08 |
| Conserved hypothetical protein | Hypothetical protein - conserved | EF1918 | -0,25 | 0,84 | **0,78** |
| Conserved hypothetical protein | Hypothetical protein - conserved | EF1926 | 0,42 | 0,75 | **-1,22** |
| Conserved hypothetical protein | Hypothetical protein - conserved | EF1949 | **-2,03** | -0,69 | 0,30 |
| Conserved hypothetical protein | Hypothetical protein - conserved | EF1968 | **0,88** | -0,78 | **-0,66** |
| Conserved hypothetical protein | Hypothetical protein - conserved | EF2018 | **1,46** | 1,15 | NA |
| Conserved domain protein | Hypothetical protein - conserved | EF2022 | 0,63 | **1,62** | -0,23 |
| Conserved hypothetical protein TIGR00048 | Hypothetical protein - conserved | EF2048 | **0,89** | -0,98 | **-2,14** |
| Conserved hypothetical protein | Hypothetical protein - conserved | EF2065 | **-1,70** | -1,27 | -0,56 |
| Conserved hypothetical protein TIGR00481 | Hypothetical protein - conserved | EF2067 | NA | **4,72** | 0,98 |
| Conserved hypothetical protein | Hypothetical protein - conserved | EF2179 | 0,37 | **-1,91** | -0,79 |
| Conserved hypothetical protein | Hypothetical protein - conserved | EF2215 | **-1,13** | 0,89 | **1,74** |
| Conserved hypothetical protein | Hypothetical protein - conserved | EF2281 | **0,99** | **1,88** | -0,29 |
| Conserved hypothetical protein | Hypothetical protein - conserved | EF2303 | 0,13 | **1,16** | -0,31 |
| Conserved hypothetical protein | Hypothetical protein - conserved | EF2306 | -0,01 | **1,32** | -0,33 |
| Conserved hypothetical protein | Hypothetical protein - conserved | EF2390 | 0,04 | 0,57 | **0,85** |
| Conserved hypothetical protein | Hypothetical protein - conserved | EF2454 | 0,49 | **1,26** | -0,11 |
| Conserved hypothetical protein | Hypothetical protein - conserved | EF2480 | -0,03 | 0,28 | **-1,15** |
| Conserved hypothetical protein | Hypothetical protein - conserved | EF2490 | **0,87** | **-0,94** | **-1,32** |
| Conserved hypothetical protein | Hypothetical protein - conserved | EF2507 | 0,25 | **2,33** | **1,10** |
| Conserved hypothetical protein | Hypothetical protein - conserved | EF2588 | 0,46 | 0,01 | **-1,16** |
| Conserved hypothetical protein | Hypothetical protein - conserved | EF2602 | -0,13 | **1,90** | 0,98 |
| Conserved hypothetical protein | Hypothetical protein - conserved | EF2621 | -0,11 | 1,13 | **1,82** |
| Conserved hypothetical protein | Hypothetical protein - conserved | EF2622 | -0,31 | 0,81 | **2,33** |
| Conserved hypothetical protein | Hypothetical protein - conserved | EF2672 | **-1,74** | -1,41 | **1,16** |
| Conserved domain protein | Hypothetical protein - conserved | EF2673 | **-1,83** | **-2,18** | 1,05 |
| Conserved hypothetical protein | Hypothetical protein - conserved | EF2678 | -0,07 | **0,96** | -0,67 |
| Conserved domain protein | Hypothetical protein - conserved | EF2697 | -0,44 | **2,50** | 0,35 |
| Conserved hypothetical protein | Hypothetical protein - conserved | EF2742 | **-0,98** | -0,57 | 0,63 |
| Conserved hypothetical protein | Hypothetical protein - conserved | EF2771 | -0,57 | **1,89** | 1,02 |
| Conserved hypothetical protein | Hypothetical protein - conserved | EF2786 | -0,32 | **1,15** | **0,95** |
| Conserved hypothetical protein | Hypothetical protein - conserved | EF2793 | -0,20 | -1,03 | **-1,31** |
| Conserved hypothetical protein | Hypothetical protein - conserved | EF2909 | **-1,11** | 0,05 | 0,54 |
| Conserved hypothetical protein | Hypothetical protein - conserved | EF2930 | 0,19 | **1,49** | 0,39 |
| Conserved hypothetical protein | Hypothetical protein - conserved | EF3021 | **-1,38** | -0,12 | 0,22 |
| Conserved hypothetical protein | Hypothetical protein - conserved | EF3055 | **-1,64** | -1,32 | -0,73 |
| Conserved hypothetical protein | Hypothetical protein - conserved | EF3151 | **-0,90** | 0,94 | 0,45 |
| Conserved hypothetical protein | Hypothetical protein - conserved | EF3155 | -0,03 | **1,17** | -0,17 |
| Conserved hypothetical protein | Hypothetical protein - conserved | EF3177 | **-1,07** | -0,43 | 0,23 |
| Conserved domain protein | Hypothetical protein - conserved | EF3259 | **0,89** | -0,16 | -0,42 |
| Conserved domain | Hypothetical protein - conserved | EF3303 | NA | 1,87 | **2,15** |
| Conserved domain protein | Hypothetical protein - conserved | EFA0074 | 0,97 | **3,61** | 0,08 |
| Structural protein, putative | Mobile and extrachromosomal element functions | EF0351 | -0,12 | **-1,59** | -0,97 |
| Transposase, putative | Mobile and extrachromosomal element functions | EF0913 | 0,81 | **1,39** | 0,33 |
| Transposase, IS256 family | Mobile and extrachromosomal element functions | EF1855 | **1,05** | 0,77 | -0,38 |
| Terminase, large subunit, putative | Mobile and extrachromosomal element functions | EF2017 | **1,25** | 0,59 | -0,17 |
| Pheromone shutdown protein TraB | Mobile and extrachromosomal element functions | EFA0002 | 0,27 | **1,06** | 0,62 |
| Replication protein | Mobile and extrachromosomal element functions | EFA0012 | 0,32 | -0,56 | **-0,94** |
| Transposase, IS6 family | Mobile and extrachromosomal element functions | EFA0016 | 0,45 | **1,72** | 0,21 |
| PemK family protein | Mobile and extrachromosomal element functions | EFA0071 | -0,87 | **2,89** | -0,39 |
| Replication-associated protein RepC | Mobile and extrachromosomal element functions | EFA0082 | **1,43** | **1,48** | -0,24 |
| Replication-associated protein RepB | Mobile and extrachromosomal element functions | EFA0083 | **2,13** | **2,79** | 0,10 |
| Replication-associated protein RepB | Mobile and extrachromosomal element functions | EFC0018 | -0,42 | **1,67** | **-1,03** |
| RepS protein, putative | Mobile and extrachromosomal element functions | EFC0019 | 0,43 | 0,99 | **0,86** |
| Ribosomal protein S6 | Protein synthesis | EF0007 | 0,14 | 0,19 | **-1,26** |
| Seryl-tRNA synthetase | Protein synthesis | EF0100 | 1,18 | -1,14 | **-1,27** |
| Ribosomal protein S12 | Protein synthesis | EF0198 | 0,21 | -0,35 | **-1,53** |
| Ribosomal protein S7 | Protein synthesis | EF0199 | 0,07 | 0,22 | **-0,86** |
| Translation elongation factor G | Protein synthesis | EF0200 | 0,08 | -0,60 | **-1,48** |
| Ribosomal protein S10 | Protein synthesis | EF0205 | -0,16 | **-1,83** | **-1,72** |
| Ribosomal protein L23 | Protein synthesis | EF0208 | 0,42 | -0,53 | **-1,76** |
| Ribosomal protein L2 | Protein synthesis | EF0209 | -0,07 | -0,39 | **-0,84** |
| Ribosomal protein S19 | Protein synthesis | EF0210 | -0,02 | -0,70 | **-1,30** |
| Ribosomal protein L22 | Protein synthesis | EF0211 | -0,03 | -0,23 | **-0,91** |
| Ribosomal protein L16 | Protein synthesis | EF0213 | 0,24 | -0,54 | **-1,27** |
| Ribosomal protein L29 | Protein synthesis | EF0214 | 0,55 | -0,25 | **-2,05** |
| Ribosomal protein S17 | Protein synthesis | EF0215 | -0,06 | -0,61 | **-1,98** |
| Ribosomal protein L24 | Protein synthesis | EF0217 | -0,12 | -0,15 | **-1,48** |
| Ribosomal protein L5 | Protein synthesis | EF0218 | 0,05 | -0,32 | **-1,56** |
| Ribosomal protein S8 | Protein synthesis | EF0220 | -0,02 | -0,27 | **-2,12** |
| Ribosomal protein L6 | Protein synthesis | EF0221 | -0,03 | -0,28 | **-1,03** |
| Ribosomal protein L18 | Protein synthesis | EF0223 | 0,12 | 0,01 | **-1,58** |
| Ribosomal protein S5 | Protein synthesis | EF0224 | 0,15 | -0,25 | **-1,90** |
| Ribosomal protein L30 | Protein synthesis | EF0225 | -0,05 | 0,02 | **-1,99** |
| Ribosomal protein L15 | Protein synthesis | EF0226 | 0,12 | 0,07 | **-1,97** |
| Translation initiation factor IF-1 | Protein synthesis | EF0229 | -0,20 | **-0,66** | **-1,68** |
| Ribosomal protein L36 | Protein synthesis | EF0230 | 0,00 | **-1,21** | **-2,26** |
| Ribosomal protein | Protein synthesis | EF0231 | 0,02 | -0,52 | **-1,46** |
| Ribosomal protein S11 | Protein synthesis | EF0232 | -0,01 | -0,40 | **-0,84** |
| Ribosomal protein L17 | Protein synthesis | EF0234 | -0,18 | **-0,99** | **-2,08** |
| Tyrosyl-tRNA synthetase | Protein synthesis | EF0633 | **1,00** | -0,66 | **-0,68** |
| Ribosomal protein L25 | Protein synthesis | EF0820 | -0,34 | **3,12** | 1,14 |
| Translation initiation factor IF-3 | Protein synthesis | EF0914 | -0,11 | 0,25 | **-1,60** |
| Ribosomal protein L35 | Protein synthesis | EF0915 | -0,03 | 0,46 | **-2,62** |
| Ribosomal protein L20 | Protein synthesis | EF0916 | -0,09 | 0,33 | **-2,84** |
| Ribosomal protein L27 | Protein synthesis | EF0970 | -0,14 | -0,12 | **-0,73** |
| Ribosomal protein L32 | Protein synthesis | EF1048 | -0,45 | **-1,45** | -0,79 |
| Phenylalanyl-tRNA synthetase, alpha subunit | Protein synthesis | EF1115 | 0,00 | **-1,62** | -0,28 |
| Ribosomal protein L31 | Protein synthesis | EF1171 | -0,05 | -0,11 | **-1,32** |
| Ribosomal protein S16 | Protein synthesis | EF1694 | -0,13 | -0,68 | **-2,39** |
| Ribosome recycling faktor | Protein synthesis | EF2395 | 0,19 | -0,68 | **-1,40** |
| Translation elongation factor Ts | Protein synthesis | EF2397 | -0,35 | -0,92 | **-1,30** |
| Ribosomal protein S2 | Protein synthesis | EF2398 | -0,08 | -0,87 | **-1,03** |
| Ribosomal protein S20 | Protein synthesis | EF2443 | -0,53 | **-1,83** | **-1,68** |
| Peptide chain release factor 1 | Protein synthesis | EF2554 | 0,11 | **-1,57** | **-1,00** |
| Ribosomal protein L7/L12 | Protein synthesis | EF2715 | -0,24 | -0,27 | **-2,29** |
| Ribosomal protein L10 | Protein synthesis | EF2716 | -0,04 | -0,61 | **-1,50** |
| Ribosomal protein L1 | Protein synthesis | EF2718 | 0,27 | 0,03 | **-1,00** |
| Ribosomal protein L11 | Protein synthesis | EF2719 | -0,21 | -0,37 | **-1,24** |
| Ribosomal protein L33 | Protein synthesis | EF2731 | **-1,05** | -0,86 | -0,68 |
| Ribosomal protein S4 | Protein synthesis | EF3070 | -0,27 | -0,68 | **-1,27** |
| Ribosomal protein L28 | Protein synthesis | EF3116 | -0,43 | **-1,42** | **-1,56** |
| Ribosomal protein S9 | Protein synthesis | EF3230 | -0,13 | -0,73 | **-1,45** |
| Ribosomal protein L34 | Protein synthesis | EF3333 | -0,39 | **-2,09** | **-1,54** |
| Chaperonin, 33 kDa | Protein fate | EF0266 | -0,36 | **-1,48** | 0,25 |
| Aminopeptidase C | Protein fate | EF0302 | -0,03 | 0,27 | **1,11** |
| Lipoate-protein ligase A | Protein fate | EF0650 | **-1,07** | -0,18 | 0,07 |
| ATP-dependent Clp protease, ATP-binding subunit ClpE | Protein fate | EF0706 | -0,29 | **1,31** | **1,76** |
| Trigger factor | Protein fate | EF0715 | -0,33 | **-0,85** | -0,66 |
| Proline dipeptidase | Protein fate | EF0973 | 0,23 | **1,55** | 0,68 |
| Lipoate-protein ligase A family protein, putativ | Protein fate | EF1144 | **-0,93** | -0,10 | 0,07 |
| Heat shock protein GrpE | Protein fate | EF1307 | **1,21** | **1,89** | 0,07 |
| DnaK protein | Protein fate | EF1308 | **1,34** | **2,30** | -0,07 |
| DnaJ protein | Protein fate | EF1310 | 0,90 | 0,93 | 0,02 |
| Heat shock protein HslV | Protein fate | EF1647 | -0,09 | 1,83 | **0,88** |
| Prolipoprotein diacylglyceryl transferase | Protein fate | EF1748 | 0,22 | **1,44** | 0,75 |
| Serine proteinase, V8 family | Protein fate | EF1817 | -0,01 | **-1,82** | **-4,43** |
| Chaperonin, 10 kDa | Protein fate | EF2634 | **1,24** | 1,05 | 0,93 |
| Oligoendopeptidase F,plasmid | Protein fate | EF2674 | 0,29 | 0,32 | **1,04** |
| Lipoate-protein ligase A | Protein fate | EF2741 | **-0,93** | -0,80 | **1,22** |
| Peptidyl-prolyl cis-trans isomerase, cyclophilin-type | Protein fate | EF2898 | -0,20 | 0,20 | **1,12** |
| Signal peptidase I | Protein fate | EF3073 | 0,52 | **1,54** | 1,03 |
| Peptidase, M16 family | Protein fate | EF3150 | -0,47 | 1,13 | **0,81** |
| Peptidase, M20/M25/M40 family | Protein fate | EF3178 | -0,26 | 0,84 | **0,99** |
| Peptidase, U32 family | Protein fate | EF3279 | **0,67** | -0,09 | 0,22 |
| ATP-dependent Clp protease, ATP-binding subunit ClpC | Protein fate | EF3282 | **-1,19** | -0,01 | 0,67 |
| Adenylosuccinate synthetase | Purines, pyrimidines, nucleosides, and nucleotides | EF0014 | 0,15 | **-2,35** | **-1,95** |
| Pur operon repressor PurR | Purines, pyrimidines, nucleosides, and nucleotides | EF0058 | 0,28 | **-2,22** | **-2,11** |
| Adenosine deaminase | Purines, pyrimidines, nucleosides, and nucleotides | EF0171 | 0,33 | **2,11** | **1,58** |
| Pyrimidin-nukleoside phosphorylase | Purines, pyrimidines, nucleosides, and nucleotides | EF0173 | **1,19** | -0,04 | -0,10 |
| Cytidine deaminase | Purines, pyrimidines, nucleosides, and nucleotides | EF0175 | **0,79** | -1,01 | -0,50 |
| Phosphopentomutase | Purines, pyrimidines, nucleosides, and nucleotides | EF0185 | -0,22 | **1,42** | 1,13 |
| Purine nucleoside phosphorylase | Purines, pyrimidines, nucleosides, and nucleotides | EF0186 | -0,22 | **0,91** | 0,57 |
| Adenylate kinase | Purines, pyrimidines, nucleosides, and nucleotides | EF0228 | 0,21 | -0,52 | **-1,82** |
| Hypoxanthine-guanine phosphoribosyltransferase | Purines, pyrimidines, nucleosides, and nucleotides | EF0264 | 0,17 | 0,77 | **0,99** |
| Dihydroorotate dehydrogenase | Purines, pyrimidines, nucleosides, and nucleotides | EF0285 | 0,12 | **-0,86** | -0,24 |
| Ribonucleoside-diphosphate reductase 2, alpha subunit | Purines, pyrimidines, nucleosides, and nucleotides | EF0471 | 0,00 | -0,04 | **1,26** |
| nrdI protein | Purines, pyrimidines, nucleosides, and nucleotides | EF0472 | 0,00 | -0,02 | **1,17** |
| Nucleoside diphosphate kinase | Purines, pyrimidines, nucleosides, and nucleotides | EF1036 | -0,03 | 0,55 | **1,41** |
| CTP synthase | Purines, pyrimidines, nucleosides, and nucleotides | EF1147 | 0,10 | **-2,07** | -0,63 |
| Adenine phosphoribosyltransferase | Purines, pyrimidines, nucleosides, and nucleotides | EF1687 | 0,12 | **-1,94** | **-1,39** |
| Orotate phosphoribosyltransferase | Purines, pyrimidines, nucleosides, and nucleotides | EF1712 | 0,04 | -0,69 | **-2,30** |
| Orotidine 5`- phosphate decarboxylase | Purines, pyrimidines, nucleosides, and nucleotides | EF1713 | 0,29 | -1,07 | **-2,53** |
| Dihydroorotate dehydrogenase | Purines, pyrimidines, nucleosides, and nucleotides | EF1714 | 0,43 | **-0,81** | **-2,82** |
| Dihydroorotate dehydrogenase electron transfer subunit | Purines, pyrimidines, nucleosides, and nucleotides | EF1715 | 0,49 | **-0,70** | **-3,30** |
| Carbamoyl-phosphate synthase, large subunit | Purines, pyrimidines, nucleosides, and nucleotides | EF1716 | 1,04 | -0,10 | **-2,27** |
| carbamoyl-phosphate synthase, small subunit | Purines, pyrimidines, nucleosides, and nucleotides | EF1717 | **1,17** | 0,42 | **-2,62** |
| Dihydroorotase | Purines, pyrimidines, nucleosides, and nucleotides | EF1718 | **1,20** | 0,71 | -1,56 |
| Aspartate carbamoyltransferase | Purines, pyrimidines, nucleosides, and nucleotides | EF1719 | **1,45** | 0,91 | **-2,23** |
| Pyrimidin operon regulatory protein PyrR | Purines, pyrimidines, nucleosides, and nucleotides | EF1721 | **1,37** | 1,04 | **-3,23** |
| Phosphoribosylaminoimidazole-succinocarboxamide synthase | Purines, pyrimidines, nucleosides, and nucleotides | EF1785 | 0,50 | -0,64 | **-3,71** |
| Deoxyguanosinetriphosphate triphosphohydrolase, putative | Purines, pyrimidines, nucleosides, and nucleotides | EF1958 | 0,92 | -0,54 | **-4,22** |
| Phosphoribosylaminoimidazole carboxylase, ATPase subunit | Purines, pyrimidines, nucleosides, and nucleotides | EF2362 | 0,55 | **1,20** | 0,53 |
| Uridylate kinase | Purines, pyrimidines, nucleosides, and nucleotides | EF2396 | 0,39 | -0,44 | **-1,18** |
| Inosine-uridine preferring nucleoside hydrolase | Purines, pyrimidines, nucleosides, and nucleotides | EF2587 | 0,23 | -0,11 | **-0,96** |
| Guanylate kinase | Purines, pyrimidines, nucleosides, and nucleotides | EF2595 | -0,30 | **1,59** | 0,22 |
| Anaerobic ribonucleoside-triphosphate reductase | Purines, pyrimidines, nucleosides, and nucleotides | EF2754 | **1,05** | 1,28 | **1,23** |
| Anaerobic ribonucleoside-triphosphate reductase activating protein | Purines, pyrimidines, nucleosides, and nucleotides | EF2755 | **1,21** | 1,02 | **1,62** |
| Transcriptional regulator, ArgR family | Regulatory functions | EF0103 | -1,54 | -0,07 | **1,16** |
| Transcriptional regulator, Crp/Fnr family | Regulatory functions | EF0107 | -0,35 | 0,39 | **2,67** |
| Transcriptional repressor CopY | Regulatory functions | EF0297 | -0,18 | **1,85** | 0,55 |
| Transcriptional regulator, DeoR family | Regulatory functions | EF0719 | -0,16 | -1,18 | **0,84** |
| DNA-binding response regulator | Regulatory functions | EF1050 | -0,09 | **-2,15** | **-0,87** |
| Sensory box histidine kinase VicK | Regulatory functions | EF1194 | -0,27 | **1,19** | **0,60** |
| Sugar-binding transcriptional regulator, LacI family | Regulatory functions | EF1240 | 0,40 | **1,38** | 0,69 |
| Heat-inducible transcription repressor HrcA | Regulatory functions | EF1306 | **1,24** | **2,07** | -0,08 |
| Transcriptional regulator, Fur family | Regulatory functions | EF1585 | -0,40 | **-1,85** | 0,08 |
| Protease synthase and sporulation negative regulatory protein pai 1, putative | Regulatory functions | EF1590 | -0,72 | -1,34 | **0,89** |
| Sucrose operon repressor ScrR | Regulatory functions | EF1604 | -0,23 | 0,87 | **0,94** |
| Transcriptional regulator CodY | Regulatory functions | EF1645 | -0,13 | 0,50 | **0,97** |
| Transcriptional regulator, GntR family | Regulatory functions | EF1676 | 0,22 | **2,08** | 0,55 |
| Transcriptional regulator, LysR familie | Regulatory functions | EF1710 | **-1,33** | -0,96 | 0,48 |
| Catabolite control protein A | Regulatory functions | EF1741 | 0,34 | -0,57 | -1,33 |
| Transcriptional regulator, SorC family | Regulatory functions | EF1965 | 0,33 | **1,31** | **1,20** |
| Transcriptional regulator, GntR family | Regulatory functions | EF2051 | **-0,80** | 0,68 | 0,24 |
| Transcriptional regulator, TetR family | Regulatory functions | EF2066 | **0,90** | **1,55** | **-1,56** |
| tspO protein, putative | Regulatory functions | EF2202 | -0,10 | **1,44** | **1,77** |
| Transcriptional regulator, TetR family | Regulatory functions | EF2203 | **-1,35** | 0,51 | 0,66 |
| Transcriptional regulator, Cro/CI family | Regulatory functions | EF2291 | -0,20 | 0,31 | **-1,90** |
| Transcriptional regulator, Cro/CI family | Regulatory functions | EF2304 | 0,32 | **1,19** | 0,22 |
| Transcriptional regulator, Cro/CI family | Regulatory functions | EF2508 | 0,16 | **1,56** | 0,17 |
| Phosphotyrosine protein phosphatase | Regulatory functions | EF3058 | 0,31 | **1,48** | 0,15 |
| Transcriptional regulator, TetR family | Regulatory functions | EF3059 | 0,34 | **1,51** | -0,08 |
| Transcriptional regulator, AbrB family | Regulatory functions | EF3261 | **-1,01** | 0,72 | 0,48 |
| DNA-binding response regulator | Regulatory functions | EF3289 | **-**0,77 | **2,16** | 0,28 |
| Sucrose operon repressor ScrR | Regulatory functions | EFA0070 | -0,15 | -0,11 | **2,02** |
| Transcriptional regulator, UvrC family | Regulatory functions | EFC0011 | 0,93 | **3,44** | 0,18 |
| PTS system, IIB component | Signal transduction | EF0019 | **-0,86** | **-2,01** | 0,12 |
| PTS system, mannose-specific IIAB components | Signal transduction | EF0020 | -0,69 | **-2,59** | -0,54 |
| PTS system, mannose-specific IIC component | Signal transduction | EF0021 | -0,46 | **-2,54** | -0,18 |
| PTS system, mannose-specific IID component | Signal transduction | EF0022 | -0,27 | **-2,24** | 0,25 |
| PTS system, fructose- specific family, IIABC components | Signal transduction | EF0717 | -0,21 | **-1,56** | **1,09** |
| PTS system, IIABC components | Signal transduction | EF0958 | **-1,63** | -1,08 | **2,25** |
| PTS system, IIB component | Signal transduction | EF1017 | **-2,35** | -2,20 | 0,42 |
| PTS system, IIC component | Signal transduction | EF1019 | **-1,90** | -0,69 | **3,00** |
| PTS system component, authentic frameshift | Signal transduction | EF1608 | **-1,19** | -0,39 | -0,06 |
| Response regulator | Signal transduction | EF1822 | -0,22 | 1,11 | **1,00** |
| PTS system, IIBC components | Signal transduction | EF2213 | -0,80 | 0,53 | **2,00** |
| Sensor histidine kinase | Signal transduction | EF3290 | -0,35 | **2,05** | 0,69 |
| PTS system, IIABC components | Signal transduction | EFA0067 | 0,17 | 1,31 | **1,12** |
| DNA-directed RNA polymerase, alpha subunit | Transcription | EF0233 | 0,08 | -0,68 | **-1,49** |
| ATP-dependent RNA helicase, DEAD/DEAH box family | Transcription | EF0846 | -0,12 | -0,88 | **-1,17** |
| Polyribonucleotide nucleotidyltransferase | Transcription | EF3064 | -0,12 | **-1,24** | -0,02 |
| DNA-directed RNA polymerase, alpha subunit, omega subunit | Transcription | EF3126 | 0,28 | 0,09 | **-0,78** |
| DNA-directed RNA polymerase, beta-prime subunit | Transcription | EF3237 | 0,27 | **-1,11** | **-0,91** |
| ABC transporter, ATP-binding protein | Transport and binding proteins | EF0017 | -0,03 | **-2,21** | **-0,99** |
| ABC transporter, permease protein | Transport and binding proteins | EF0057 | 0,04 | **-1,94** | -0,20 |
| Major facilitator family transporter | Transport and binding proteins | EF0082 | -0,55 | **-3,17** | **-4,45** |
| Formate/nitrite transporter family protein | Transport and binding proteins | EF0094 | **1,35** | **-2,86** | **-3,46** |
| C4-dicarboxylate transporter, putative | Transport and binding proteins | EF0108 | -0,12 | 0,60 | **2,36** |
| ABC transporter, ATP-bindingprotein | Transport and binding proteins | EF0178 | 0,74 | **-1,59** | **-0,93** |
| ABC transporter, permease protein | Transport and binding proteins | EF0179 | 0,55 | **-2,53** | **-1,29** |
| ABC transporter, permease protein | Transport and binding proteins | EF0180 | 0,50 | **-2,31** | **-1,24** |
| Amino acid ABC transporter, amino acid-binding/permease protein | Transport and binding proteins | EF0247 | -0,18 | **-1,21** | -0,39 |
| Potassium-transporting ATPase, subunit B | Transport and binding proteins | EF0568 | 0,28 | 0,66 | **2,38** |
| Potassium-transporting ATPase, subunit C | Transport and binding proteins | EF0569 | NA | -0,21 | **1,20** |
| Cation ABC transporter, permease protein | Transport and binding proteins | EF0576 | 0,23 | **1,55** | -0,46 |
| Amino acid permease family protein | Transport and binding proteins | EF0635 | **1,29** | **-4,13** | **-1,80** |
| Na+/H+ antiporter | Transport and binding proteins | EF0636 | **1,63** | **-3,04** | **-1,18** |
| Mat efflux family protein | Transport and binding proteins | EF0660 | 0,61 | **2,16** | **1,44** |
| Glycine betaine/carnitine/choline ABC transporter, ATP-binding protein | Transport and binding proteins | EF0674 | **-1,44** | -0,51 | 0,60 |
| Amino acid ABC transporter, ATP-binding protein | Transport and binding proteins | EF0760 | -0,29 | **-2,01** | -0,27 |
| Cation efflux family protein | Transport and binding proteins | EF0859 | 0,24 | 0,79 | **2,94** |
| Glycine betaine/carnitine/choline ABC transporter, permease protein | Transport and binding proteins | EF0862 | -0,07 | **1,11** | **1,23** |
| Glycine betaine/carnitine/choline ABC transporter, Glycine betaine/carnitine/choline -binding protein | Transport and binding proteins | EF0863 | -0,11 | **1,43** | **1,33** |
| Glycine betaine/carnitine/choline ABC transporter, permease protein | Transport and binding proteins | EF0864 | -0,29 | **1,37** | **1,34** |
| Glycine betaine/carnitine/choline transporter, ATP-binding protein | Transport and binding proteins | EF0865 | -0,17 | **1,38** | 1,07 |
| Cation-transporting ATPase, E1-E2 family | Transport and binding proteins | EF0871 | -0,47 | **2,03** | 0,66 |
| Amino acid ABC transporter, ATP-binding protein | Transport and binding proteins | EF0892 | 0,29 | **-2,12** | -0,66 |
| Peptide ABC transporter, peptide-binding protein | Transport and binding proteins | EF0907 | -0,26 | **2,51** | **0,75** |
| Amino acid permease family protein | Transport and binding proteins | EF0929 | **0,83** | -0,23 | -0,13 |
| Aquaporin Z | Transport and binding proteins | EF1192 | 0,13 | -0,89 | **­-1,43** |
| Permease domain protein | Transport and binding proteins | EF1321 | 0,00 | 0,70 | **1,09** |
| ABC transporter, ATP-binding/permease protein | Transport and binding proteins | EF1341 | 0,33 | **1,89** | 0,79 |
| Sugar ABC transporter, permease protein | Transport and binding proteins | EF1343 | **-1,44** | 0,22 | **1,89** |
| Sugar ABC transporter, permease protein | Transport and binding proteins | EF1344 | -1,45 | -0,40 | **2,15** |
| Sukker ABC transporter, sugar-binding protein | Transport and binding proteins | EF1345 | **-1,37** | **-1,10** | **2,42** |
| ABC transporter, ATP-binding protein | Transport and binding proteins | EF1675 | 0,74 | **1,95** | 0,29 |
| Phosphate-binding protein | Transport and binding proteins | EF1705 | 0,61 | **1,70** | 0,11 |
| Uracil permease | Transport and binding proteins | EF1720 | **1,32** | **1,00** | **-3,02** |
| Phosphate ABC transporter, ATP-binding protein | Transport and binding proteins | EF1756 | **0,72** | **-1,03** | -0,26 |
| Cation-transporting ATPase, E1-E2 family | Transport and binding proteins | EF1938 | -0,59 | -0,59 | **1,15** |
| Amino acid permease familie protein | Transport and binding proteins | EF2047 | -0,14 | **-1,65** | -0,62 |
| ABC transporter, permease protein, putative | Transport and binding proteins | EF2049 | 0,12 | **5,19** | 0,20 |
| ABC transporter, ATP-binding protein | Transport and binding proteins | EF2050 | -0,69 | **4,88** | -0,20 |
| ABC transporter, ATP-binding protein | Transport and binding proteins | EF2074 | -1,15 | **2,20** | -0,04 |
| ABC transporter, permease protein | Transport and binding proteins | EF2075 | -0,65 | **3,56** | -0,21 |
| ABC transporter, permease protein | Transport and binding proteins | EF2081 | 0,94 | -0,34 | **-2,37** |
| ABC transporter, ATP-binding protein | Transport and binding proteins | EF2153 | 0,79 | **-1,22** | -0,33 |
| Xanthine permease | Transport and binding proteins | EF2364 | **1,52** | 0,51 | -0,20 |
| ABC transporter, ATP-binding protein | Transport and binding proteins | EF2394 | 0,13 | **1,12** | 0,63 |
| ABC transporter, permease protein | Transport and binding proteins | EF2485 | 0,74 | **-1,54** | **-2,32** |
| ABC transporter, ATP-binding protein | Transport and binding proteins | EF2486 | 0,73 | -1,19 | **-2,12** |
| Cadmium-translocating P-type ATPase | Transport and binding proteins | EF2623 | 0,06 | **2,24** | **3,27** |
| Glycine betaine/L-proline ABC transporter, ATP-binding subunit | Transport and binding proteins | EF2641 | -0,21 | **3,42** | **2,92** |
| Glycine betaine/L-proline ABC transporter, glycine betaine/L-proline-binding/permease protein | Transport and binding proteins | EF2642 | 0,69 | **4,99** | **4,21** |
| ABC transporter, ATP-binding protein | Transport and binding proteins | EF2720 | -0,68 | 1,08 | **1,17** |
| ABC transporter, ATP-binding/permease protein | Transport and binding proteins | EF2920 | 0,52 | **1,46** | 0,28 |
| Xanthine/uracil permeases family protein | Transport and binding proteins | EF2935 | -0,36 | **-1,34** | -0,19 |
| Ribose uptake protein, putative | Transport and binding proteins | EF2959 | -0,27 | 1,29 | **1,16** |
| ABC transporter, ATP-binding protein | Transport and binding proteins | EF2986 | -0,16 | **3,10** | -0,83 |
| Sulfate transporter familiy/STAS domain protein | Transport and binding proteins | EF3004 | 1,03 | -0,71 | **-1,42** |
| Sodium:dicarboxylate symporter family protein | Transport and binding proteins | EF3022 | **0,86** | -1,02 | -1,01 |
| Formate/nitrite transporter family protein | Transport and binding proteins | EF3069 | 0,55 | **-1,66** | **-2,90** |
| Iron compound ABC transporter, permease protein | Transport and binding proteins | EF3085 | 0,08 | **1,79** | 0,08 |
| ABC transporter, permease protein | Transport and binding proteins | EF3199 | **-2,04** | -1,07 | 1,22 |
| Oxidoreductase, short chain dehydrogenase/reductase family | Unknown function | EF0076 | **-**0,84 | 0,49 | **2,22** |
| Diacylglycerol kinase catalytic domain protein | Unknown function | EF0090 | **-1,37** | -0,83 | 0,84 |
| LysM domain protein | Unknown function | EF0443 | -0,03 | **-**0,76 | **2,55** |
| AMP-binding family protein | Unknown function | EF0452 | **1,26** | -0,34 | **-1,36** |
| OsmC/Ohr family protein | Unknown function | EF0453 | **-2,16** | 0,47 | **1,73** |
| Decarboxylase, putative | Unknown function | EF0634 | 0,82 | **-2,13** | **-1,65** |
| Glyoxalase family protein | Unknown function | EF0666 | **-1,21** | -0,31 | 0,41 |
| Acyltransferase, putative | Unknown function | EF0783 | -0,67 | **1,53** | 0,70 |
| Pentapeptide repeterende family protein | Unknown function | EF0905 | 0,38 | **1,83** | **0,90** |
| Acetyltransferase, GNAT family | Unknown function | EF0945 | **0,57** | **2,60** | **1,07** |
| Endonuclease/exonuclease/phosphatase family protein | Unknown function | EF0960 | **-1,45** | -0,62 | **2,32** |
| Oxidoreductase, Gfo/Idh/MocA family | Unknown function | EF1008 | -0,36 | 0,00 | **0,93** |
| Hydrolase, haloacid dehalogenase-like family | Unknown function | EF1039 | -0,38 | **1,57** | 0,21 |
| Hexapeptide-repeat containing-acetyltransferase | Unknown function | EF1066 | **-0,84** | -0,18 | **1,14** |
| Oxidoreductase, putative | Unknown function | EF1108 | **0,62** | **1,57** | **1,32** |
| YkgG family protein | Unknown function | EF1110 | 0,26 | 0,43 | **0,78** |
| Oxidoreductase, aldo/keto reductase family | Unknown function | EF1138 | -0,87 | -0,01 | **1,09** |
| Glutamine amidotransferase, class I | Unknown function | EF1139 | **-1,08** | **-1,74** | -0,09 |
| DegV family protein | Unknown function | EF1191 | -0,44 | **-1,35** | **-0,96** |
| Oxidoreductase, putative | Unknown function | EF1226 | **1,75** | -0,43 | **-1,1** |
| S1 RNA binding domain protein | Unknown function | EF1312 | -0,32 | -0,01 | 0,05 |
| BadF/BadG/BcrA/BcrD ATPase family protein | Unknown function | EF1327 | **1,56** | -0,08 | -0,29 |
| Dihydroxyacetone kinase family protein | Unknown function | EF1361 | **-1,26** | -0,55 | 1,08 |
| LysM domain protein | Unknown function | EF1546 | **-0,82** | 0,22 | **0,42** |
| GTPase, putative | Unknown function | EF1549 | 0,25 | -0,76 | **-0,75** |
| YitT family protein | Unknown function | EF1555 | -0,59 | -0,25 | **0,67** |
| CoA-binding domain protein | Unknown function | EF1616 | **-0,99** | -0,43 | 0,27 |
| lacX protein, putative | Unknown function | EF1644 | -0,22 | 0,62 | **1,27** |
| Glucose-inhibited division protein | Unknown function | EF1649 | -0,39 | -2,44 | **1,57** |
| Oxidoreductase, zinc-binding | Unknown function | EF1671 | **-1,47** | -0,13 | 0,27 |
| DegV family protein, putative | Unknown function | EF1684 | **-0,71** | 0,70 | 0,57 |
| KH domain protein | Unknown function | EF1693 | -0,22 | -0,06 | **-1,73** |
| Aminotransferase, class I | Unknown function | EF1706 | 0,41 | **1,40** | 0,37 |
| GTP-binding protein | Unknown function | EF1916 | **-0,84** | **-1,82** | 0,80 |
| Coenzyme F420 hydrogenase domain protein | Unknown function | EF2014 | **-1,54** | 0,74 | 0,35 |
| Glyoxylase family protein | Unknown function | EF2214 | **-1,22** | **1,20** | **1,83** |
| Aminotransferase, class V | Unknown function | EF2392 | 0,10 | **1,02** | **0,67** |
| GTP-binding protein TypA | Unknown function | EF2460 | -0,38 | **-1,31** | -0,71 |
| HD domene protein | Unknown function | EF2470 | **-0,96** | 0,37 | **-0,72** |
| GcvH family protein | Unknown function | EF2500 | 0,03 | **2,27** | 0,23 |
| Sua5/YciO/YrdC/YwlC family protein | Unknown function | EF2552 | 0,31 | **-1,02** | **-0,46** |
| DNA-binding protein, putative | Unknown function | EF2638 | **-2,16** | -0,58 | 0,77 |
| Diacylglycerol kinase catalytic domain protein | Unknown function | EF2644 | **-1,00** | -1,05 | 0,82 |
| Hydrolase, haloacid dehalogenase-like family | Unknown function | EF2681 | **0,83** | 0,38 | -0,42 |
| Oxidoreductase, Gfo/Idh/MocA family | Unknown function | EF2734 | -0,09 | **1,60** | **0,96** |
| ErfK/YbiS/YcfS/YnhG family protein, putative | Unknown function | EF2860 | 0,03 | **2,00** | **1,42** |
| D-isomer specific 2-hydroxyacid dehydrogenase family protein | Unknown function | EF2901 | **-1,37** | 0,28 | **1,85** |
| Hydrolase, haloacid dehalogenase-like family | Unknown function | EF2927 | 0,05 | 0,57 | **1,07** |
| DNA-binding protein, putativ | Unknown function | EF2933 | 1,08 | 0,36 | **-1,09** |
| Acetyltransferase, GNAT family | Unknown function | EF3079 | 0,42 | -0,05 | **1,00** |
| YitT family protein | Unknown function | EF3091 | **0,85** | -0,59 | **-0,87** |
| Glyoxalase family protein | Unknown function | EF3092 | -0,30 | -0,58 | **0,90** |
| Thiamine pyrophosphokinase family protein | Unknown function | EF3117 | **0,73** | 0,86 | 0,22 |
| Hydrolase, haloacid dehalogenase-like family | Unknown function | EF3158 | 0,01 | 0,49 | **1,69** |
| LrgB family protein | Unknown function | EF3193 | **1,57** | **0,78** | **-0,57** |
| Oxidoreductase, pyridine nucleotide-disulfide family | Unknown function | EF3257 | **0,73** | **-2,56** | **-2,41** |
| PemI family protein | Unknown function | EFA0072 | -0,64 | **2,35** | -0,11 |
